# Prepaid parameter estimation without likelihoods

**DOI:** 10.1371/journal.pcbi.1007181

**Published:** 2019-09-09

**Authors:** Merijn Mestdagh, Stijn Verdonck, Kristof Meers, Tim Loossens, Francis Tuerlinckx

**Affiliations:** KU Leuven, University of Leuven, Leuven, Belgium; Oberlin College, UNITED STATES

## Abstract

In various fields, statistical models of interest are analytically intractable and inference is usually performed using a simulation-based method. However elegant these methods are, they are often painstakingly slow and convergence is difficult to assess. As a result, statistical inference is greatly hampered by computational constraints. However, for a given statistical model, different users, even with different data, are likely to perform similar computations. Computations done by one user are potentially useful for other users with different data sets. We propose a pooling of resources across researchers to capitalize on this. More specifically, we preemptively chart out the entire space of possible model outcomes in a prepaid database. Using advanced interpolation techniques, any individual estimation problem can now be solved on the spot. The prepaid method can easily accommodate different priors as well as constraints on the parameters. We created prepaid databases for three challenging models and demonstrate how they can be distributed through an online parameter estimation service. Our method outperforms state-of-the-art estimation techniques in both speed (with a 23,000 to 100,000-fold speed up) and accuracy, and is able to handle previously quasi inestimable models.

## Author summary

Interesting nonlinear models are often analytically intractable. As a result, statistical inference has to rely on massive, time-intensive, simulations. The main idea of our method is to avoid the redundancy of similar computations that typically occur when different researchers independently fit the same model to their particular dataset. Instead, we propose to pool computational resources across the researchers interested in any given model. The prepaid method starts with an extensive simulation of datasets across the parameter space. The simulated data are compressed into summary statistics, and the relation to the parameters is learned using machine learning techniques. This results in a parameter estimation machine that produces accurate estimates very quickly (a 23,000 to 100,000-fold speed up compared to traditional methods).

This is a *PLOS Computational Biology* Methods paper.

## Introduction

Models without an analytical likelihood are increasingly used in various disciplines, such as genetics [1], ecology [2, 3], economics [4, 5] and neuroscience [6]. For such models, parameter estimation is a major challenge for which a variety of solutions have been proposed [2, 1, 7]. All these methods have in common that they rely on extensive Monte Carlo simulations and that their convergence can be painstakingly slow. As a result, the current methods can be very time consuming.

To date, the practice is to analyse each data set separately. However, considering all the calculations that have ever been performed during parameter estimation of a particular type of model, for each different data set, one cannot help but notice an incredible waste of resources. Indeed, simulations performed while estimating one data set may also be relevant for the estimation of another. Currently, each researcher estimating the same model with different data will start from scratch, and can not benefit from all the possibly relevant calculations that have already been performed in earlier analyses by other researchers, in other locations, on different hardware, and for other data sets, but concerning the same model.

Hence, we propose an estimation scheme that dramatically increases overall efficiency by avoiding this immense redundancy. Most current algorithms are inherently iterative and (slowly) adjust their window of interest to the area of convergence. Instead, we propose to generate an all-inclusive and one-shot prepaid database that is capable of estimating the parameters of a particular model for all potential data sets and with almost no additional computation time per data set. Our approach starts with the extensive simulation of data sets across the entire parameter space. These data are then compressed into summary statistics, after which the relation between the summary statistics and the parameters can be learned using interpolation techniques. Finally, global optimization methods can be used on the previously created (hence, prepaid) database for accurate and fast parameter estimation on any device. This results in a mass lookup and interpolation scheme that can produce estimates to any given dataset very quickly.

**Fig 1:**
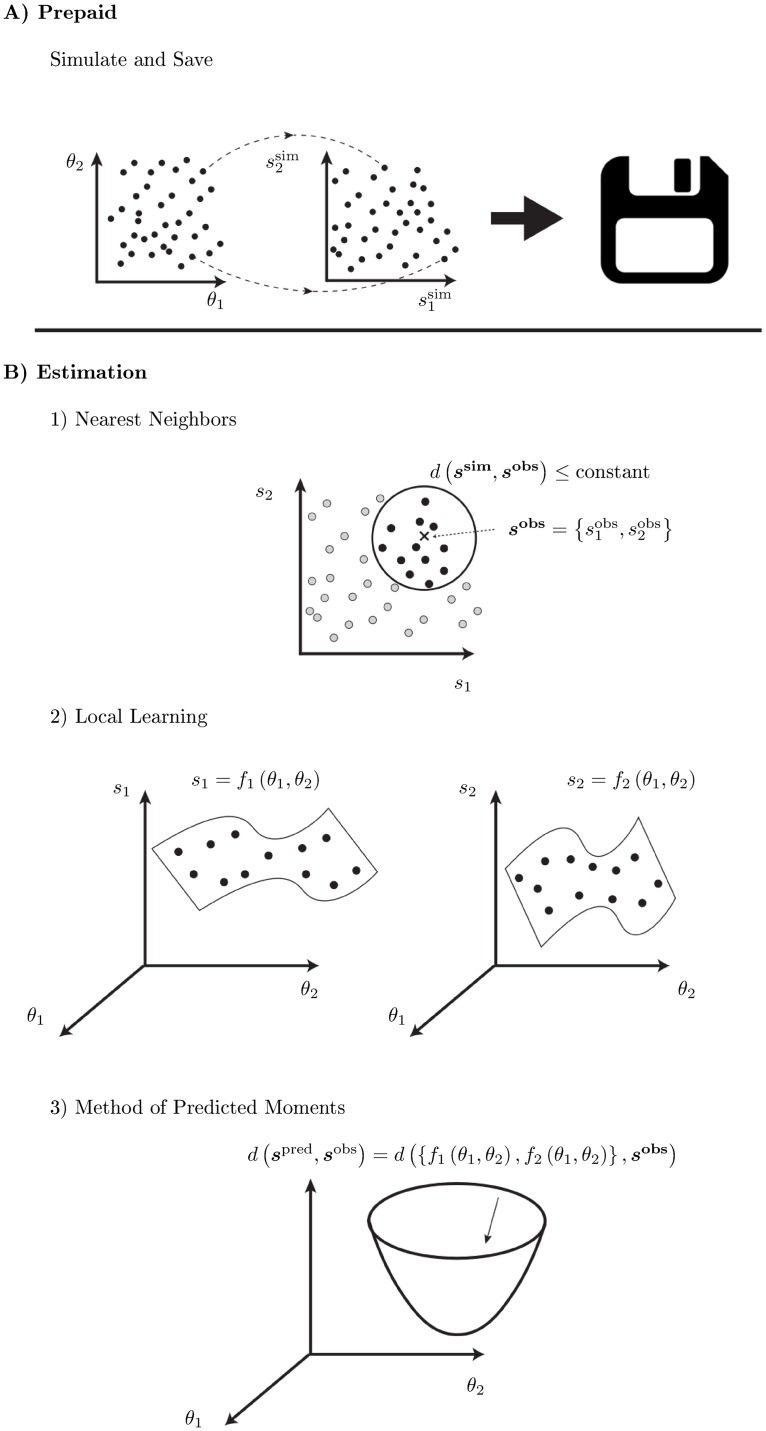
Graphical illustration of the prepaid parameter estimation method.

In Fig 1 we present a graphical illustration of the prepaid parameter estimation method. First (panel A), for a sufficient number of parameter vectors ***θ***, large data sets are simulated, compressed into summary statistics (i.e., ***s***^sim^) and saved—creating the prepaid grid. This prepaid grid is computed beforehand and the results are stored at a central location. Second (panel B1), the observed (data) summary statistics (***s***^obs^) are compared to the simulated (data) summary statistics (i.e., ***s***^sim^) using an appropriate objective loss function *d*(***s***^sim^, ***s***^obs^) and a number of nearest neighbor simulated summary statistics are selected. The loss function is related to the loss function used in the generalized method of moments [8] and method of simulated moments [9].

Third (panel B2), interpolation methods are used to find the relation ***s*** = *f*(***θ***) between the parameter values and the summary statistics for the selected points of the previous step [10, 11]. In this paper, we use tuned least squares support vector machines, LS-SVM [12]. Finally (panel B3), the objective loss function *d*(***s***^pred^, ***s***^obs^), now using predicted summary statistics ***s***^pred^, is minimized as a function of the unknown parameter values using an optimizer.

A number of important aspects of the prepaid method deserve special mention. First, the parameter space is required to be bounded. If this is unnatural for a given parametrization, then the parameters have to be appropriately transformed to a bounded space. Second, we typically start from a uniform distribution of parameter vectors in the final parameter space. This choice reflects on the uniformity of the grid’s resolution, but has no further implications provided the grid is sufficiently dense. Bayesian priors can be implemented without recreating the prepaid grid, since the prior can be taken into account in the loss function. Third, often a user is not interested in a single instance of a model, but rather has data from several experimental conditions that share some common parameters but assume other ones to be different. Also in these cases the prepaid grid does not need to be recreated, as the parameter constraints can be included through priors with tuning parameters (i.e., penalties). Fourth, the creation of the prepaid database is a fixed cost and usually takes from a couple of hours to one or more days, depending on the complexity of the model of interest (see below for a number of examples). Once its prepaid database is created, the parameters of the model can be estimated for any data set, with any amount of data (number of observations).

The prepaid method can be studied theoretically in simple situations. For example, in Methods, we apply the prepaid idea for estimating the mean of a normal distribution and study some of its properties for two different summary statistics. In what follows, the prepaid method will be applied to three more complicated, realistic scenarios.

## Results

### Example 1: The Ricker model

In a first example, we apply our prepaid method to the Ricker model [13, 2] which describes the dynamics of the number of individuals *y*_*t*_ in a species over time (with *t* = 1 to *T*_obs_):

$$\begin{array}{c} \begin{array}{cc} y_{t} & ∼\text{Pois}(\phi N_{t})\\ N_{t+1} & =rN_{t}e^{-N_{t}+e_{t}} \end{array} \end{array}$$ (1)

where $e_{t}∼N(0,\sigma^{2})$. The variables *N*_*t*_ (i.e., the expected number of individuals at time *t*) and *e*_*t*_ are hidden states. Given an observed time series ${\left\{y_{t}\right\}}_{t=1}^{T_{\text{obs}}}$, we want to estimate the parameters ***θ*** = {*r*, *σ*, *ϕ*}, where *r* is the growth rate, *σ* the process noise and *ϕ* a scaling parameter. The Ricker model can demonstrate near-chaotic or chaotic behavior and no explicit likelihood formula is available.

Wood [2] used the synthetic likelihood to estimate the model’s parameters. In the original synthetic likelihood approach (denoted as SL^Orig^), the assumed multivariate normal distribution of the summary statistics is used to create a synthetic likelihood. The mean and covariance matrix of this normal distribution are functions of the unknown parameters and are calculated using a large number of model simulations. The synthetic likelihood is proportional to the posterior distribution from which is sampled using MCMC and a posterior mean is computed.

Wood’s synthetic likelihood SL^Orig^ approach is compared to the prepaid method, where we create a prepaid grid of the mean and the covariance matrix of a similar set of summary statistics. Prepaid estimation comes in multiple variants, depending on the use of an interpolation method. The first, which uses only the prepaid grid points and chooses the nearest neighbor (maximum synthetic likelihood) as final estimate, will be called ${\text{SL}}_{\text{ML}}^{\text{Grid}}$. The second, ${\text{SL}}_{\text{ML}}^{\text{SVM}}$, uses LS-SVM to interpolate between the parameters in the prepaid grid to increase accuracy. The differential evolution algorithm (a global optimizer; [14]) is used to maximize this interpolated synthetic (log)likelihood. Additional details on the implementation of the synthetic likelihood can also be found in Methods.

**Fig 2:**
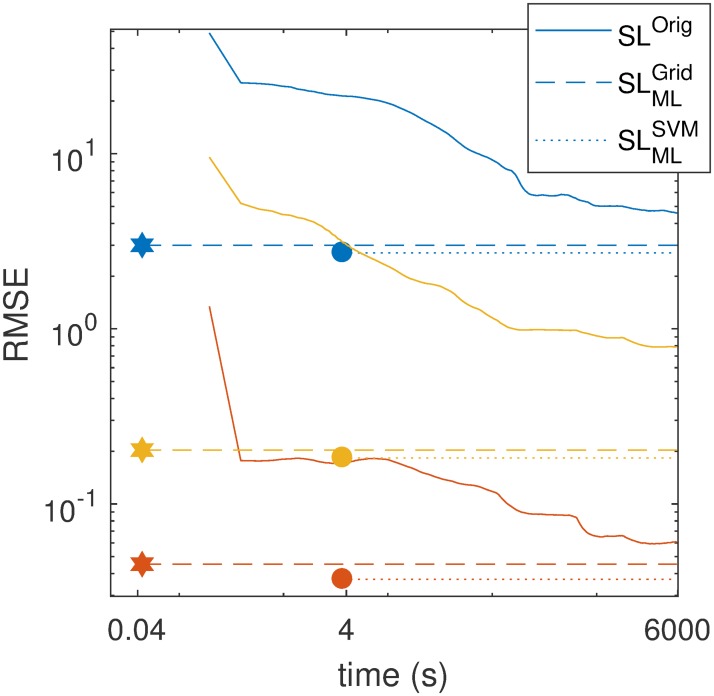
The RMSE versus the time needed for the estimation of the three parameters of the Ricker model (see Eq 1). The RMSE and time are based on 100 test data sets with *T*_obs_ = 1000. The three colors represent the three parameters (blue for *r*, red for *σ* and yellow for *ϕ*). Solid lines represent the SL^Orig^ approach, dashed lines the ${\text{SL}}_{\text{ML}}^{\text{Grid}}$ approach (using only nearest neighbors), and dotted lines the ${\text{SL}}_{\text{ML}}^{\text{SVM}}$ approach (using interpolation). The stars and the dots represent the time needed for the ${\text{SL}}_{\text{ML}}^{\text{Grid}}$ and the ${\text{SL}}_{\text{ML}}^{\text{SVM}}$ estimation, respectively. The estimates for SL^Orig^ are posterior means, based on the second half of the finished MCMC iterations. The time of the prepaid method shown in this picture does not include the creation of the prepaid grid, but only the time needed for any researcher to estimate the parameters once a prepaid grid is available.

Fig 2 shows both the accuracy of parameter recovery (as measured with the RMSE) and computation time for the three methods under comparison: (1) SL^Orig^ as in [2], the prepaid method (2) with interpolation (${\text{SL}}_{\text{ML}}^{\text{SVM}}$), and (3) without (${\text{SL}}_{\text{ML}}^{\text{Grid}}$) interpolation. As can be seen in Fig 2, the prepaid estimation techniques lead to better results than the synthetic likelihood for *T*_obs_ = 1, 000, both in accuracy and speed. The SL^Orig^ method leads to some clear outliers (see Methods) which testifies to possible convergence problems (probably due to local minima). The prepaid method suffers much less from this problem. Most striking is the speed up of the prepaid method: The ${\text{SL}}_{\text{ML}}^{\text{Grid}}$ version of the prepaid estimation is finished before a single iteration of the 30,000 iterations in the synthetic likelihood method has been completed—100,000 times faster. In addition, it is demonstrated that the coverages of the prepaid method confidence intervals are very close or exactly equal to the nominal value (we look at 95% bootstrap-based confidence intervals). SVM interpolation is mainly helpful for large *T*_obs_, where one expects a higher accuracy of the estimates and the grid is too coarse. The analyses with large *T*_obs_ could only be completed in a reasonable time using the prepaid method (See Methods for more detailed information).

In the application above, the tacitly assumed prior on the parameter space is uniform. In addition, there is only one data set for which a single triplet of parameters (*r*, *σ*, *ϕ*) needs to be estimated. In Methods, we show how both limitations can be relaxed. First, it is explained how different priors for the Ricker model can be implemented. Second, it is discussed what can be done if there are two data sets (i.e., conditions) for which it holds that *r*_1_ = *r*_2_ and *σ*_1_ = *σ*_2_ but *ϕ*_1_ and *ϕ*_2_ are not related.

Finally, we also tested our estimation process on the population dynamics of the Chilo partellus, extracted from Fig 1 in Taneja and Leuschner [15, 16]. Here we found that *r* = 1.10 (95% confidence interval 1.06–1.34), *σ* = 0.43 (95% confidence interval 0.30–0.54) and *ϕ* = 140.60 (95% confidence interval = 43.94–208.19). We found similar results using the synthetic likelihood method (see Methods), but our estimation was 4000 times faster.

### Example 2: A stochastic model of community dynamics

A second example we use to illustrate the prepaid inference method is a trait model of community dynamics [17] used to model the dispersion of species. For this model (see also Methods section), there are four parameters to be estimated: *I*, *A*, *h*, and *σ*. As with the first application, there is no analytical expression for the likelihood [17].

As an established benchmark procedure for this trait model, we apply the widely used Approximate Bayesian Computation (ABC) method [18, 19, 20, 21] as implemented in the Easy ABC package and denoted here as ${\text{ABC}}_{\text{PM}}^{\text{Orig}}$ (PM stands for posterior means, which will be used as point estimates) [22]. As priors, we use uniform distributions on bounded intervals for log(*I*), log(*A*), *h* and log(*σ*) (see Methods for the exact specifications), but this can be easily changed as explained for the first example.

To allow for a direct comparison with the ABC method (${\text{ABC}}_{\text{PM}}^{\text{Orig}}$), and to illustrate the versatility of the prepaid method, we have also implemented three Bayesian versions of the prepaid method. The first, ${\text{SL}}_{\text{PM}}^{\text{Grid}}$, creates a posterior proportional to the prepaid synthetic likelihood. The second method, ${\text{ABC}}_{\text{PM}}^{\text{Grid}}$, saves not only the mean and covariance matrix of the summary statistics for every parameter in the prepaid grid, but also a large set of uncompressed summary statistics. Using these statistics we are able to approximate an ABC approach. The third, ${\text{ABC}}_{\text{PM}}^{\text{SVM}}$, again interpolates between the grid points to achieve a higher accuracy.

All methods result in accuracies of the same order of magnitude as can be seen in Table 1. The main difference is again the speed of the methods: ${\text{ABC}}_{\text{PM}}^{\text{Grid}}$ is about 23,000 times faster than traditional ABC. For small sample sizes, all ABC based methods achieve good coverage. However, for large sample sizes, ${\text{ABC}}_{\text{PM}}^{\text{Orig}}$ cannot be used anymore (because of the unduly long computation time). For the prepaid versions and large samples, it is necessary to use SVM interpolation between the grid points to get accurate results.

**Table 1:** The RMSE of the estimates of the test set of the trait model. *T*_obs_ refers to the number of observations (i.e., vector with species frequencies) and Ω is the number of prepaid points.

*T*_obs_	version	Ω	log(*I*)	log(*A*)	*h*	log(*σ*)
1	${\text{ABC}}_{\text{PM}}^{\text{Orig}}$	/	0.17	0.67	7.45	0.74
1	${\text{SL}}_{\text{PM}}^{\text{Grid}}$	100000	0.17	0.66	7.49	0.7
1	${\text{ABC}}_{\text{PM}}^{\text{Grid}}$	100000	0.16	0.63	7.9	0.7
1	${\text{ABC}}_{\text{PM}}^{\text{Grid}}$	500000	0.16	0.62	8.17	0.7
1000	${\text{ABC}}_{\text{PM}}^{\text{Grid}}$	100000	0.07	0.35	6.41	0.61
1000	${\text{ABC}}_{\text{PM}}^{\text{Grid}}$	500000	0.05	0.27	4.83	0.48
1000	${\text{ABC}}_{\text{PM}}^{\text{SVM}}$	100000	0.03	0.23	5.24	0.42
1000	${\text{ABC}}_{\text{PM}}^{\text{SVM}}$	500000	0.03	0.21	4.39	0.4

### Example 3: The Leaky Competing Accumulator for choice response times

In a third example, we apply our method to stochastic accumulation models for elementary decision making. In this paradigm, a person has to choose, as quickly and accurately as possible, the correct response given a stimulus (e.g., is a collection of points moving to the left or to the right). Task difficulty is manipulated by applying different levels of stimulus ambiguity.

A popular neurally inspired model of decision making is the Leaky Competing Accumulator (LCA [23]). For two response options, two noisy evidence accumulators (stochastic differential equations, see Methods section) race each other until one of them reaches the required amount of evidence for the corresponding option to be chosen. The time that is required to reach that option’s threshold is interpreted as the associated choice response time. For different levels of stimulus difficulty, the model produces different levels of accuracy and choice response time distributions. The evidence accumulation process leading up to these choices and response times is assumed to be indicative of the activation levels of neural populations involved in the decision making.

As in the first two examples, there is no analytical likelihood available that can be used to estimate the parameters of the LCA. Moreover, the LCA is an extremely difficult model to estimate. To the best of our knowledge, only [24] systematically investigated the recovery of the LCA parameters, but for a slightly different model (with three choice options) and with a method that is impractically slow for very large sample sizes, making it difficult to show near-asymptotic recovery properties with.

For an experiment with four stimulus difficulty levels, the LCA model has nine parameters. However, after a reparametrization of the model (but without a reduction in complexity), it is possible to reduce the prepaid space to four dimensions (see Methods) and conditionally estimate the remaining subset of the parameters with a less computationally intensive method. Three variants of the prepaid method have been implemented: taking the nearest neighboring parameter set (based on a symmetrized *χ*^2^ distance between distributions) on the prepaid grid (${\text{CHISQ}}_{\text{NN}}^{\text{Grid}}$), averaging over the grids nearest neighboring parameter sets of 100 non-parametric bootstrap samples (${\text{CHISQ}}_{\text{BS}}^{\text{Grid}}$), using SVM interpolation for every bootstrap estimate (${\text{CHISQ}}_{\text{BS}}^{\text{SVM}}$). A nearest neighbor or bootstrap averaged estimate completes in about a second on a Dell Precision T3600 (4 cores at 3.60GHz), an SVM interpolated estimate requires a couple of minutes extra.

**Fig 3:**
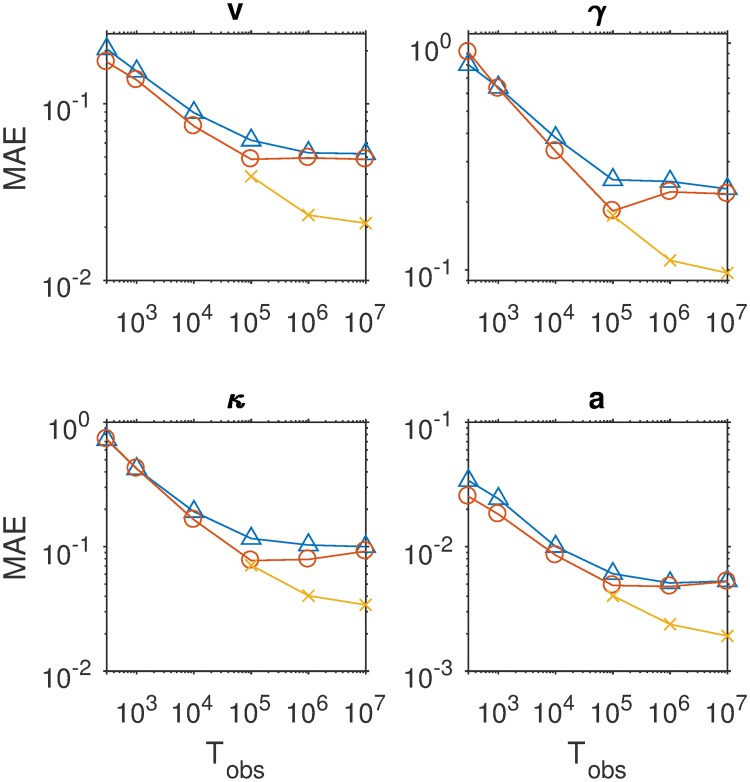
The mean absolute error of the estimates of four central parameters of the LCA (common input *v*, leakage *γ*, mutual inhibition *κ*, evidence threshold *a*) as a function of sample size (abscissa) and for three different methods: (1) choosing the nearest neighbor grid point in the space of summary statistics (${\text{CHISQ}}_{\text{NN}}^{\text{Grid}}$, triangles); (2) using the average of a set of nearest neighbor grid points based on bootstrap samples (${\text{CHISQ}}_{\text{BS}}^{\text{Grid}}$, open circles) and (3) using SVM interpolation between the 100 nearest neighbors (${\text{CHISQ}}_{\text{BS}}^{\text{SVM}}$, crosses).

**Fig 4:**
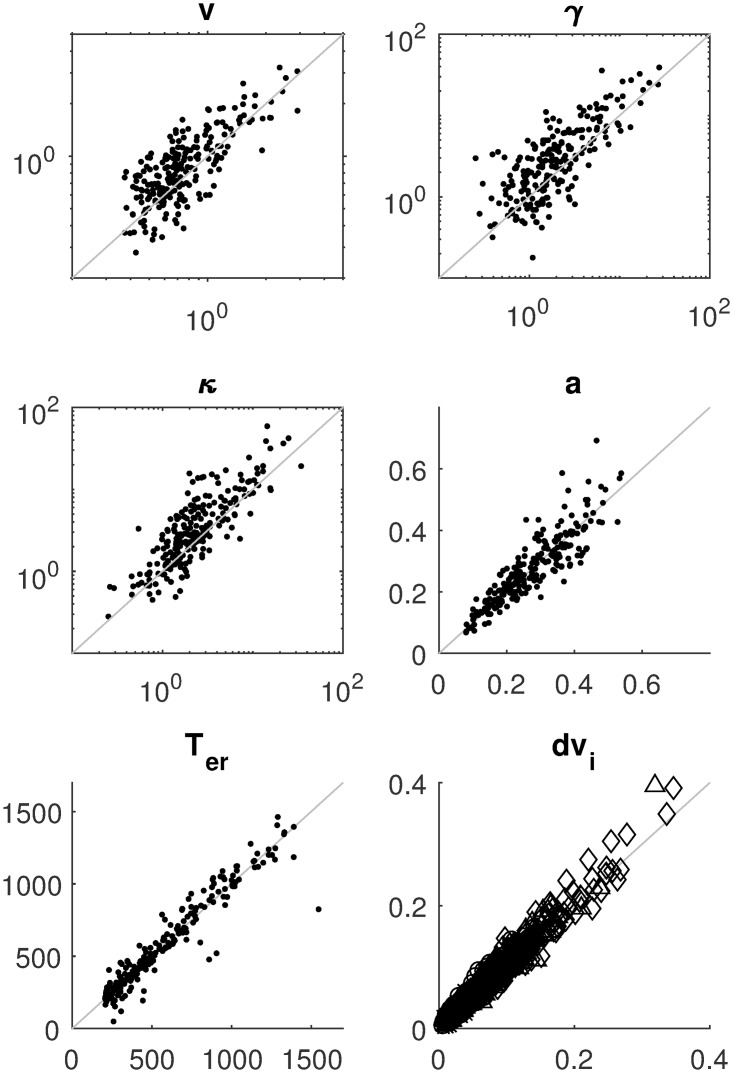
Parameter recovery for the LCA model with 1200 observations (300 in each of the four difficulty conditions); the true value on the abscissa and estimated value on the ordinate. The same parameters as in Fig 3 are shown. The method used to produce these estimates is the averaged bootstrap approach (${\text{CHISQ}}_{\text{BS}}^{\text{Grid}}$, see Methods for details).

Fig 3 displays the mean absolute error (MAE) of the estimates for four of the nine parameters as a function of sample size, separately for three estimation methods. The results for the other parameters are similar and can be consulted in the Methods section. It can be seen that with increasing sample size, MAE decreases. The SVM method pays off especially for larger samples. Fig 4 shows detailed recovery scatter plots for a subset of the parameters for 1,200 observed trials, which is the typical size of decision experiments. To get better recovery, larger sample sizes have to be considered (see Methods section). In general, recovery is much better than what has been reported in [24]. The coverage of the method, based on non-parametric bootstrapping, is satisfactory for all sample sizes, provided SVM interpolated estimates are used for *T*_obs_ > 100000. In addition, we do not find evidence for a fundamental identification issue with the two option LCA, as has been stated in [24].

## Discussion

In three examples, we have demonstrated the efficacy and versatility of the prepaid method. The prepaid method is at least as accurate as current methods, but many times faster (23,000 to 100,000-fold speed up). Besides the improvements at the level of speed and accuracy, the prepaid method has a number of other distinct advantages. First, the prepaid method can be used for a very large number of observations, contrary to the synthetic likelihood or ABC methods. The use of very large simulated data sets allows a practical investigation of large-sample properties of the estimator, which is a problem for the synthetic likelihood and ABC. Second, because of the enormous speed improvement and having data sets available across the whole parameter space, the prepaid method allows for fast yet extensive testing of recovery of simulated data across this space—the recovery of every single parameter set can be evaluated. Such a practice leads to detailed internal quality control of the used estimation algorithm.

Although the idea behind the prepaid method is fairly simple, we want to anticipate a few misconceptions that might arise. First, as has been demonstrated in the context of the Ricker model (the first example), the prepaid method can easily deal with different priors and with equality constraints on parameters, without the need to recreate the underlying prepaid grid. Second, the observed data based on which the model parameters have to be estimated can be of any size, again without the need to recreate the prepaid grid for each and every sample size.

In the first two examples the synthetic likelihood [2] is used, but its exact effect on likelihood based model selection techniques, such as information criteria, is not known. For users interested in model selection, we propose cross-validation as its implementation is straight forward. The main draw-back of this resampling method, its computational burden, is mitigated by the use of the prepaid method.

Ideally, the prepaid databases and the corresponding estimation algorithms will be constructed and made available by a team of experts for the model at hand. Subsequently, a cloud based service can be set up to offer high quality model estimations to a broad public of researchers. As an example, we created such a service for the Ricker model in Eq 1: http://www.prepaidestimation.org/, where we allow the user to estimate the parameters of the Ricker model for personal data as well as 4 example data sets including one real life data set [15, 16]. By using such a cloud based service, researchers that need their data analyzed with computationally challenging models, can avoid many of the pitfalls they would otherwise encounter venturing out on their own. This practice will also lead to increased reproducibility of computational results.

As the need for reproducibility and transparency is (fortunately) increasingly recognized by the broader scientific community, critical model users will want to see proof of robust estimation across the entire parameter space, and be able to test this themselves. The current standard of simply sharing the code of a procedure, still grants developers of complex models/methods a layer of protection from public scrutiny, because the level of knowledge and infrastructure required to check the work is considerable and not many are called to take up the challenge. The prepaid method, however, allows any user with a basic grasp of statistics to check the consistency of the model and method, using data they have simulated themselves. In the future, we expect a natural evolution towards a situation where stakeholders in certain models (the developers and/or heavy users) will provide an estimation service or outsource this endeavor to a third party. The infrastructure required for hosting such a service is orders of magnitude lighter than what is required for the calculation of the database itself or a thorough simulation study for that matter. We are currently hosting the Ricker model on a very modest system (medium level desktop).

A first possible objection to the prepaid method is the considerable initial simulation cost (for the examples discussed, prepaid simulations took up to a couple of days on a 20-core processor). However, this overhead cost will dissipate entirely as increasingly more estimates are sourced from the same prepaid database. Moreover, the initial prepaid cost can be easily distributed across multiple interested parties. Further, because the database can be used for internal quality control, additional simulation studies investigating the recovery of parameters are made redundant. Indeed, whenever a new model and associated parameter estimation method are proposed, a recovery study is needed to study how well the parameters of the model can be estimated using the method. When such a simulation study is set up in a rigorous way, the prepaid grid will have been (partially or completely) constructed. For the first and the second example, the time to create the prepaid grid was of the same order as that of the parameter recovery study included for the estimation techniques the prepaid grid was compared with. Note however that the parameter recovery study of the traditional techniques was only partial, as data sets with more observations, for which the parameter estimation would take an excessively long time using only traditional methods, were excluded. If those would be included, a parameter recovery study would be at least 10 times slower than the creation of the prepaid grid. The fact that a parameter recovery study takes at least as much time as the creation of the prepaid grid makes sense. A recovery study should test the estimation of parameters in the whole realm of possible data sets. The prepaid grid exactly covers this realm.

The argumentation above shows that a parameter recovery study and a prepaid grid are very related. In fact, Jabot, saw the necessity of reusing ABC simulations to reduce computation time in his recovery study for the model of the second example [17]. More broadly, we are convinced that other researchers also have used similar tricks to avoid redundant simulation within their own research context. For example, a reviewer of this manuscript noted that s/he uses a prepaid grid (although not named so) when trying models in which the parameters change across trials. The main difference with prepaid estimation is that we propose to reuse these simulations to facilitate future estimations.

A second possible objection is that the prepaid grid, unsurprisingly, does not escape the curse of dimensionality: The grid size grows exponentially with the number of parameters. The prepaid method is most effective for highly nonlinear models with substantively meaningful parameters, as they appear in various computational modeling fields. For these models, all simulation based estimation techniques struggle with the curse of dimensionality. For the prepaid method, this limitation can be alleviated in a number of ways. First, the use of interpolation techniques allows for a substantial reduction of the number of prepaid points (by a factor of five for the same accuracy in the trait model example; see Methods section). Second, as is shown in the LCA example, it is possible to only partially apply the prepaid method and combine it with traditional estimation techniques. In this way, the less challenging parameters can be estimated conditionally on a prepaid grid of the more intricately connected ones. Third, as shown by tackling three challenging examples, current storage and/or memory technology can accommodate realistically sized prepaid databases.

A last possible objection is the risk, that once the prepaid grid is created for a certain model, researchers will be biased towards using this particular model. They may prefer the relatively easy prepaid estimation of this model over the use of other models without a prepaid grid. We hope however that also the creation of the prepaid grid is manageable enough for any model to prevent such scenarios.

A possible improvement of the prepaid method lies in a smarter construction of the prepaid grid. First, there is a straightforward theoretical angle: spreading the grid points out according to Jeffrey’s prior rather than a naïve parameter based prior, would lead to a more evenly distributed estimation accuracy, and therefore a smaller database size will suffice for a given minimum accuracy. Additionally, the database could be improved based on the actual queries of users. If the simulation grid proves a bit thin around the requested area (not a lot of unique grid points), more grid points can be added there. This way more detail is added where it matters.

Finally, the prepaid method also offers exciting opportunities for future research. First, another typical case where the same model has to be estimated multiple times, arises in a multilevel context (where several individual analyses are regularized by a set of hyperparameters defined on the group). Although extremely useful, multilevel analyses typically come with an additional computational burden. Because the synthetic likelihood, as any likelihood, can be extended to a multilevel context, the prepaid method should be too. Further research is needed to develop this idea.

Second, the prepaid philosophy can also be used to choose a good set of summary statistics, which are necessary for simulation based estimation techniques. During the creation of the prepaid grid many summary statistics can be saved, with no additional simulation cost. The effectiveness of combinations of summary statistics are then easily tested in parameter recovery studies as the prepaid estimation is so quick.

It is our strong belief that this method will massively democratize the use of many computationally expensive models, which are now reserved for people with access to specific high-end hardware (e.g., GPUs, HPC). Apart from such democratization, this approach could significantly impact the current work flow of scientific modeling, in which every part of the estimation is carried out locally by an individual researcher.

## Methods

### A toy example: Estimating the mean of a normal

For a very simple setting, we want to study the performance of the prepaid methods analytically.

Assume *y*_*i*_ ∼ *N*(*μ*, *s*^2^) (*i* = 1, …, *T*_obs_) with the mean *μ* unknown (and to be estimated and the standard deviation *s* known (so number of parameters *K* = 1). The observed mean is denoted as $\bar{y}$. We will explore two situations. In the first situation, $\bar{y}$ will be our summary statistic *s*^obs^ (hence number of summary statistics *R* = 1) to estimate *μ* ($\bar{y}$ is also a sufficient statistic for *μ*). In the second situation, we will study what happens if $s^{\text{obs}}={\bar{y}}^{2}$ is chosen to be the summary statistic.

#### Situation 1: $s^{\text{obs}}=\bar{y}$

As a prepaid grid, we take *N*_*r*_ evenly spaced *μ*-values with spacing or gap size Δ = *μ*_*j*+1_ − *μ*_*j*_ (see Fig 1, left figure of panel A; in our case the parameter space is one dimensional). For each value *μ*_*j*_, *T*_sim_ values of *y* are simulated and the sample average is computed (i.e., ${\bar{y}}_{j}^{\text{sim}}$) (see middle figure of panel A in Fig 1). Typically, *T*_sim_ = 1000 or larger. Hence, every value of *μ*_*j*_ is paired with a particular ${\bar{y}}_{j}^{\text{sim}}$: $\left(\mu_{j},{\bar{y}}_{j}^{\text{sim}}\right)$.

Given an observed $\bar{y}$, the *N* nearest neighbors of simulated statistics ${\bar{y}}_{j}^{\text{sim}}$ are selected: $\left(\mu_{\left(1\right)},{\bar{y}}_{\left(1\right)}^{\text{sim}}\right)$, $\left(\mu_{\left(2\right)},{\bar{y}}_{\left(2\right)}^{\text{sim}}\right),\ldots ,\left(\mu_{\left(N\right)},{\bar{y}}_{\left(N\right)}^{\text{sim}}\right)$ (see panel B1 of Fig 1), such that $|{\bar{y}}_{\left(1\right)}^{\text{sim}}-\bar{y}\left|\leq \right|{\bar{y}}_{\left(2\right)}^{\text{sim}}-\bar{y}\left|\leq \cdots \leq \right|{\bar{y}}_{\left(N\right)}^{\text{sim}}-\bar{y}|$. Typically, *N* = 100. In principle, the selected *μ*s depend on $\bar{y}$, but for simplicity we suppress this dependence in the notation.

Because of the linearity of the problem, we can safely assume that if *T*_sim_ is large enough, the *N* selected *μ* values are all consecutive or nearly consecutive (because of noise in the prepaid simulation of ${\bar{y}}^{\text{sim}}$, it can happen that the *N* selected *μ* values are not consecutive). We denote the average of these *N* *μ*-values as *M*_*μ*_. Ordering all values from smallest to largest (denoting the *j*th value as *μ*_[*j*]_ and assuming they are exactly consecutive, *M*_*μ*_ can be expressed as):

$$\begin{array}{cc} M_{\mu }^{\bar{y}} & =\frac{1}{N}\;\sum_{j=1}^{N}\;\mu_{\left[j\right]}\\ & =\frac{1}{N}\;\sum_{j=0}^{N-1}\;(\mu_{\left[1\right]}+j\;\Delta )\\ & =\mu_{\left[1\right]}+\frac{\Delta }{N}\;\sum_{j=1}^{N-1}\;j\\ & =\mu_{\left[1\right]}+\frac{\Delta (N-1)}{2} \end{array}$$

where we have defined *μ*_[1]_ as

$$\begin{array}{c} \mu_{\left[1\right]}\equiv \min_{i\;\in \;1,2,...,N}(\mu_{\left[i\right]}). \end{array}$$

In addition (assuming that all values are exactly consecutive), their variance *V*_*μ*_ is given by

$$\begin{array}{cc} V_{\mu }^{\bar{y}} & =\frac{1}{N}(\sum_{j=1}^{N}\;\mu_{\left(j\right)}^{2})-M_{\mu }^{\bar{y}2}\\ & =\frac{1}{N}\;(\sum_{j=0}^{N-1}\;{\left(\mu_{\left(1\right)}+j\;\Delta \right)}^{2})-M_{\mu }^{\bar{y}2}\\ & =\frac{1}{N}\;(\sum_{j=0}^{N-1}\;(\mu_{\left(1\right)}^{2}+2j\;\Delta \;\mu_{\left(1\right)}+j^{2}\;\Delta^{2}))-M_{\mu }^{\bar{y}2}\\ & =\mu_{\left(1\right)}^{2}+\frac{2\;\Delta \;\mu_{\left(1\right)}}{N}\;(\sum_{j=1}^{N-1}\;j)+\frac{\Delta^{2}}{N}\;(\sum_{j=1}^{N-1}\;j^{2})-M_{\mu }^{\bar{y}2}\\ & =\mu_{\left(1\right)}^{2}+\Delta \;\mu_{\left(1\right)}\;(N-1)+\frac{\Delta^{2}\;(N-1)(2N-1)}{6}-M_{\mu }^{\bar{y}2}\\ & =\frac{\Delta^{2}\;(N-1)(2N-1)}{6}-\frac{\Delta^{2}{\left(N-1\right)}^{2}}{4}\\ & =\frac{\Delta^{2}\;\left(N-1\right)\left(N+1\right)}{12}\\ & \approx \frac{\Delta^{2}\;N^{2}}{12}. \end{array}$$

Hence, their standard deviation is $S_{\mu }\approx \frac{\Delta N}{2\sqrt{3}}$ and thus independent of $\bar{y}$.

Using the *N* nearest neighbour pairs, we assume as a linear interpolator (see panel B2 of Fig 1) in this example a linear regression model that links the simulated statistics to the true underlying *μ*: ${\bar{y}}_{j}^{\text{sim}}=\beta_{0}+\beta_{1}\mu_{j}+\epsilon_{j}$, with $\epsilon_{j}∼N(0,\frac{s^{2}}{T_{\text{sim}}})$. Obviously, *β*_0_ = 0 and *β*_1_ = 1.

Given $\bar{y}$, *N* selected prepaid points and the fitted linear regression model, we know from linear regression theory that:

$$\begin{array}{c} \left(\begin{array}{c} {\hat{\beta }}_{0}\\ {\hat{\beta }}_{1} \end{array}\right)∼N_{2}((\begin{array}{c} 0\\ 1 \end{array}),(\begin{array}{cc} \sigma_{0}^{2} & \sigma_{01}\\ \sigma_{01} & \sigma_{1}^{2} \end{array})), \end{array}$$

where 0 and 1 are the true *β*_0_ and *β*_1_ and

$$\begin{array}{cc} \sigma_{0}^{2} & =\text{Var}\left({\hat{\beta }}_{0}|\bar{y}\right)\approx \frac{s^{2}}{T_{\text{sim}}}(\frac{1}{N}+\frac{12M_{\mu }^{\bar{y}2}}{\Delta^{2}N^{3}})\\ \sigma_{1}^{2} & =\text{Var}\left({\hat{\beta }}_{1}|\bar{y}\right)\approx \frac{s^{2}}{T_{\text{sim}}}\frac{12}{\Delta^{2}N^{3}}\\ \sigma_{01} & =\text{Cov}\left({\hat{\beta }}_{0},{\hat{\beta }}_{1}|\bar{y}\right)=-M_{\mu }\sigma_{1}^{2}\approx -\frac{s^{2}}{T_{\text{sim}}}\frac{12M_{\mu }^{\bar{y}}}{\Delta^{2}N^{3}}. \end{array}$$

The distribution is assumed to hold for repeated simulations of the replicated statistics in the prepaid grid.

Because we work with linear regression, the optimization problem is simple. In this case, the optimal value of *μ* for a given $\bar{y}$ can be found by inverting the regression line:

$$\begin{array}{c} \hat{\mu }=\frac{\bar{y}-{\hat{\beta }}_{0}}{{\hat{\beta }}_{1}}. \end{array}$$

In this simple example, the method of predicted moments from Panel B3 in Fig 1 yields an exact solution for the estimated mean, given the observed sample average.

Next, we can study the properties of $\hat{\mu }$. We begin by calculating the conditional mean $E(\hat{\mu }|\bar{y})$ and conditional variance $\text{Var}(\hat{\mu }|\bar{y})$. Hence, we treat the observed data (or sample average) as given and fixed. These expectations are taken over different simulations of ${\bar{y}}_{j}^{\text{sim}}$’s in the prepaid grid. Before giving the expressions, it is useful to note that

$$\begin{array}{c} \left(\begin{array}{c} \bar{y}-{\hat{\beta }}_{0}\\ {\hat{\beta }}_{1} \end{array}\right)∼N_{2}((\begin{array}{c} \bar{y}\\ 1 \end{array}),(\begin{array}{cc} \sigma_{0}^{2} & -\sigma_{01}\\ -\sigma_{01} & \sigma_{1}^{2} \end{array})). \end{array}$$

Now, using the approximations given in [25] for ratios of random variables, we find that:

$$\begin{array}{cc} E(\hat{\mu }|\bar{y}) & =E(\frac{\bar{y}-{\hat{\beta }}_{0}}{{\hat{\beta }}_{1}}|\bar{y})\\ & \approx \frac{E(\bar{y}-{\hat{\beta }}_{0}|\bar{y})}{E({\hat{\beta }}_{1}|\bar{y})}-\frac{1}{E{\left({\hat{\beta }}_{1}|\bar{y}\right)}^{2}}\text{Cov}\left(\bar{y}-{\hat{\beta }}_{0},{\hat{\beta }}_{1}|\bar{y}\right)+\frac{E(\bar{y}-{\hat{\beta }}_{0}|\bar{y})}{E{\left({\hat{\beta }}_{1}|\bar{y}\right)}^{3}}\text{Var}\left({\hat{\beta }}_{1}|\bar{y}\right)\\ & \approx \frac{\bar{y}}{1}-\frac{1}{1^{2}}\frac{s^{2}}{T_{\text{sim}}}\frac{12M_{\mu }^{\bar{y}}}{\Delta^{2}N^{3}}+\frac{\bar{y}}{1^{3}}\frac{s^{2}}{T_{\text{sim}}}\frac{12}{\Delta^{2}N^{3}}\\ & =\bar{y}(1+\frac{s^{2}}{T_{\text{sim}}}\frac{12}{\Delta^{2}N^{3}})-\frac{M_{\mu }^{\bar{y}}}{T_{\text{sim}}}\frac{12s^{2}}{\Delta^{2}N^{3}} \end{array}$$

and

$$\begin{array}{cc} \text{Var}(\hat{\mu }|\bar{y}) & =\text{Var}(\frac{\bar{y}-{\hat{\beta }}_{0}}{{\hat{\beta }}_{1}}|\bar{y})\\ & \approx \frac{E{\left(\bar{y}-{\hat{\beta }}_{0}|\bar{y}\right)}^{2}}{E{\left({\hat{\beta }}_{1}|\bar{y}\right)}^{2}}(\frac{\text{Var}(\bar{y}-{\hat{\beta }}_{0}|\bar{y})}{E{\left(\bar{y}-{\hat{\beta }}_{0}|\bar{y}\right)}^{2}}+\frac{\text{Var}({\hat{\beta }}_{1}|\bar{y})}{E{\left({\hat{\beta }}_{1}|\bar{y}\right)}^{2}}-\frac{2\text{Cov}(\bar{y}-{\hat{\beta }}_{0},{\hat{\beta }}_{1}|\bar{y})}{E\left(\bar{y}-{\hat{\beta }}_{0}|\bar{y}\right)E\left({\hat{\beta }}_{1}|\bar{y}\right)})\\ & =\frac{{\bar{y}}^{2}}{1^{2}}(\frac{\sigma_{0}^{2}}{{\bar{y}}^{2}}+\frac{\sigma_{1}^{2}}{1^{2}}-\frac{2(-\sigma_{01})}{\bar{y}\cdot 1})\\ & =\sigma_{0}^{2}+{\bar{y}}^{2}\sigma_{1}^{2}-2\bar{y}M_{\mu }^{\bar{y}}\sigma_{1}^{2}\\ & \approx \frac{s^{2}}{T_{\text{sim}}N}(1+\frac{12M_{\mu }^{\bar{y}2}+12{\bar{y}}^{2}-24\bar{y}M_{\mu }^{\bar{y}}}{\Delta^{2}N^{2}})\\ & =\frac{s^{2}}{T_{\text{sim}}N}(1+\frac{12{\left(M_{\mu }^{\bar{y}}-\bar{y}\right)}^{2}}{\Delta^{2}N^{2}}). \end{array}$$

Invoking the double expectation theorem to arrive at the unconditional expectations, we have:

$$\begin{array}{cc} E(\hat{\mu }) & =E[E(\hat{\mu }|\bar{y})]\\ & \approx E\left(\bar{y}\right)(1+\frac{s^{2}}{T_{\text{sim}}}\frac{12}{\Delta^{2}N^{3}})-\frac{E(M_{\mu }^{\bar{y}})}{T_{\text{sim}}}\frac{12s^{2}}{\Delta^{2}N^{3}}\\ & =\mu (1+\frac{s^{2}}{T_{\text{sim}}}\frac{12}{\Delta^{2}N^{3}})-\frac{E(M_{\mu }^{\bar{y}})}{T_{\text{sim}}}\frac{12s^{2}}{\Delta^{2}N^{3}}\\ & =\mu -\frac{\alpha }{T_{\text{sim}}}\frac{12s^{2}}{\Delta^{2}N^{3}}, \end{array}$$ (2)

where $\alpha =E(M_{\mu }^{\bar{y}})-\mu$, that is, the difference between the expected value of the mean of the selected nearest neighbors *μ*’s and the true *μ*. Likewise, we can derive the marginal variance $\text{Var}(\hat{\mu })$. We will assume that the variance in $M_{\mu }^{\bar{y}}$ is equal to $\text{Var}\left(\bar{y}\right)=\frac{s^{2}}{T_{\text{obs}}}$. In addition, we assume that $M_{\mu }^{\bar{y}}$ and $\bar{y}$ correlate perfectly, such that $\text{Cov}\left(M_{\mu }^{\bar{y}},\bar{y}\right)=\text{Var}\left(\bar{y}\right)$. For this particular example, these assumptions make sense. Then we can derive that:

$$\begin{array}{cc} \text{Var}(\hat{\mu }) & =E[\text{Var}(\hat{\mu }|\bar{y})]+\text{Var}[E(\hat{\mu }|\bar{y})]\\ & \approx \frac{s^{2}}{T_{\text{sim}}N}(\frac{12\left(\frac{s^{2}}{T_{\text{obs}}}+\mu^{2}\right)+12E\left(M_{\mu }^{\bar{y}2}\right)-24E\left(M_{\mu }^{\bar{y}}\right)\mu }{\Delta^{2}N^{2}}+1)+\frac{s^{2}}{T_{\text{obs}}}(1+\frac{12s^{2}}{T_{\text{sim}}\Delta^{2}N^{3}}+\frac{144s^{4}}{T_{\text{sim}}^{2}\Delta^{4}N^{6}})\\ & =\frac{s^{2}}{T_{\text{obs}}}+\frac{12s^{4}}{T_{\text{obs}}T_{\text{sim}}\Delta^{2}N^{3}}+\frac{144s^{6}}{T_{\text{obs}}{T_{\text{sim}}}^{2}\Delta^{4}N^{6}}+\frac{s^{2}}{T_{\text{sim}}N}+\frac{12s^{2}\left(\frac{s^{2}}{T_{\text{obs}}}+\mu^{2}\right)+12s^{2}E\left(M_{\mu }^{\bar{y}2}\right)-24s^{2}E\left(M_{\mu }^{\bar{y}}\right)\mu }{T_{\text{sim}}\Delta^{2}N^{3}}\\ & =\frac{s^{2}}{T_{\text{obs}}}+\frac{s^{2}}{T_{\text{sim}}N}+\frac{12s^{4}}{T_{\text{sim}}T_{\text{obs}}\Delta^{2}N^{3}}+\frac{144s^{6}}{T_{\text{sim}}^{2}T_{\text{obs}}\Delta^{4}N^{6}}+\frac{12s^{2}}{T_{\text{sim}}\Delta^{2}N^{3}}(\frac{s^{2}}{T_{\text{obs}}}+E\left({\left(\mu -M_{\mu }^{\bar{y}}\right)}^{2}\right))\\ & =\frac{s^{2}}{T_{\text{obs}}}+\frac{s^{2}}{T_{\text{sim}}N}+\frac{12s^{2}E\left({\left(\mu -M_{\mu }^{\bar{y}}\right)}^{2}\right)}{T_{\text{sim}}\Delta^{2}N^{4}}+\frac{24s^{4}}{T_{\text{sim}}T_{\text{obs}}\Delta^{2}N^{3}}+\frac{144s^{6}}{T_{\text{sim}}^{2}T_{\text{obs}}\Delta^{4}N^{6}} \end{array}$$ (3)

**Fig 5:**
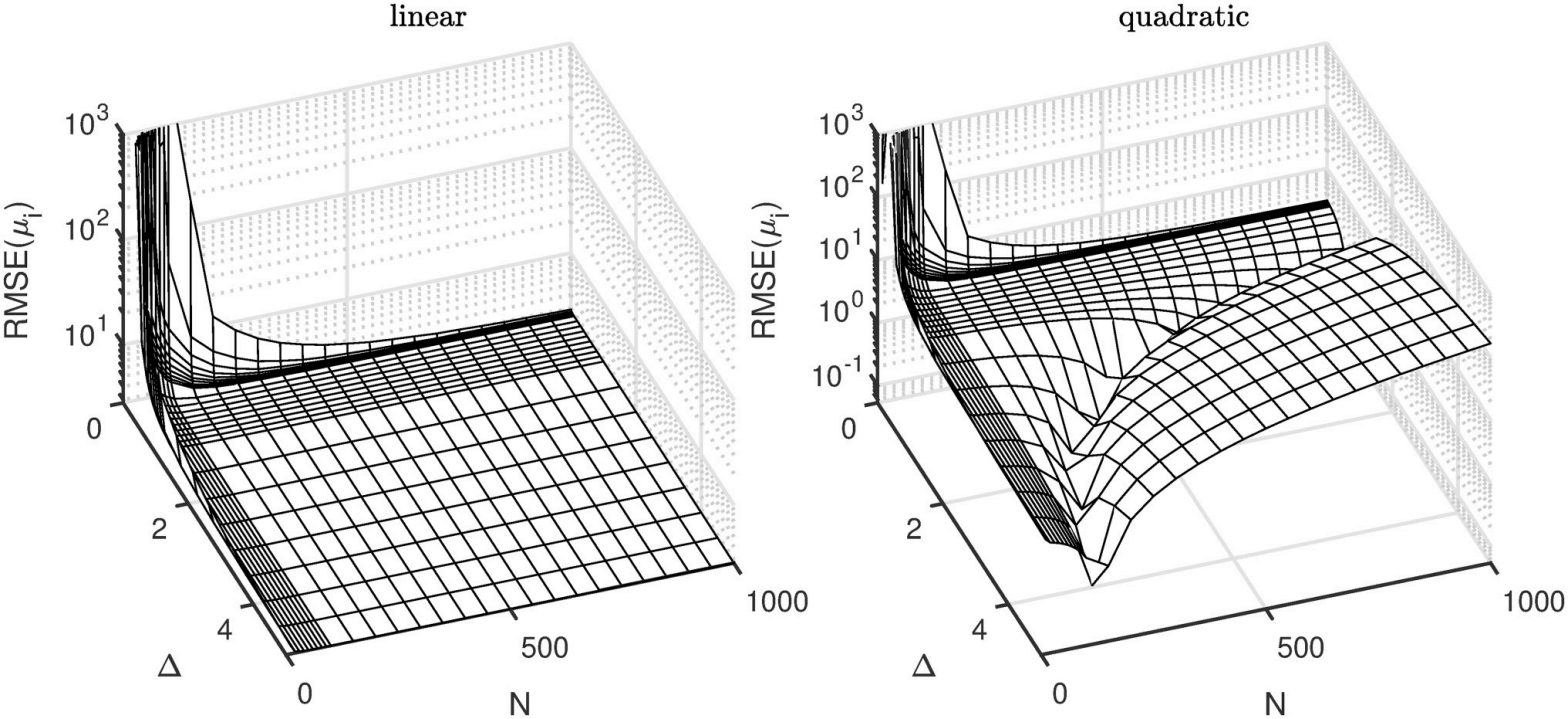
RMSE (based on a simulation study) of the toy example estimation as function of the gap size (Δ) and number of nearest neighbors selected to carry out the interpolation (*N*). The left panel is called situation 1 in which $s^{\text{obs}}=\bar{y}$ and the right panel is situation 2 ($s^{\text{obs}}={\bar{y}}^{2}$). For the second situation, the trade-off between Δ and *N* is clearly visible.

From Eq 2, we learn that if there is no systematic deviation in the selection of *μ*-grid points, the prepaid estimator is unbiased. In the other case, the bias decreases with *T*_sim_ but is proportional to *s*^2^. In Eq 3, the leading term of the variance is $\frac{s^{2}}{T_{\text{obs}}}$, which is the same as in classical estimation theory. For the other terms, they all have *T*_sim_ (or a power of it) in the denominator. Because *T*_sim_ is usually quite large, these terms tend to be in general of lesser importance. However, some terms also have both *N* (the number of selected nearest neighbor grid points) and Δ (the gap size) in the denominator. It is worthwhile to note that increasing the resolution (i.e., decreasing Δ), while keeping *N* constant, will increase the additional terms and thus add to the error. The reason for this is that the interpolation is defined on a too small grid, leading to uncertainty in the estimated regression. This effect is illustrated in the left panel of Fig 5 in which the root mean square error (RMSE) is shown for the estimation of *μ* for different values of *N* and Δ. The plot is constructed by means of a simulation study, but confirms our analytical results.

#### Situation 2: $s^{\text{obs}}={\bar{y}}^{2}$

In the second situation, we will again estimate *μ* (the unknown mean of a unit variance normal), but in this case $s^{\text{obs}}={\bar{y}}^{2}$ is used as a statistic. Thus, the relation between the simulated statistics ${\bar{y}}^{{\text{sim}}^{2}}$ and *μ* is quadratic (and thus nonlinear). Again we use a local linear approximation. Clearly, this approximation will only be approximately valid if we do not choose the area of approximation too large. However, unlike in the first situation, we do expect an additional effect of the approximation error.

No analytical derivations were made for this case, but we conducted a similar simulation study as in situation 1. The results (in terms of RMSE) are shown in the right panel of Fig 5. As can be seen, there is a clear optimality trade-off visible between Δ and *N*. This can be explained as follows: Fix *N* and then consider the gap size Δ. If Δ is too small, we get a similar phenomenon as in the left panel, that is a large RMSE. However, if we take Δ too large, then the approximation error will dominate (because the linear interpolation misfits the quadratic relation). The optimal point will be different for different *N*.

This toy example demonstrates the sound theoretical foundations of the prepaid method in well-behaved situations. However, the question is how well the method performs for real life examples.

### Application 1: The Ricker model

The basic model equations of the Ricker model is given in Eq 1.

#### Synthetic likelihood estimation

For the synthetic likelihood estimation (SL^Orig^), we made use of the synlik package [26]. The synthetic likelihood *l*_*s*_ for a data set with summary statistics ***s***^obs^ and a certain parameter vector ***θ*** = (*r*, *σ*, *ϕ*) is given by

$$\begin{array}{c} l_{s}\left(\theta \right)=-\frac{1}{2}{\left(s^{\text{obs}}-{\hat{\mu }}_{\theta }\right)}^{T}{\hat{\Sigma }}_{\theta }^{-1}(s^{\text{obs}}-{\hat{\mu }}_{\theta })-\frac{1}{2}\log|{\hat{\Sigma }}_{\theta }|, \end{array}$$ (4)

where ${\hat{\mu }}_{\theta }$ and ${\hat{\Sigma }}_{\theta }$ are the estimated mean and covariance of the summary statistics when Eq 1 is simulated multiple times with parameter ***θ***.

The statistics used by the synthetic likelihood function were the average population size, the number of zeros, the autocovariances up to lag 5, the coefficients of the quadratic linear autoregression of $y_{t}^{0.3}$ and the coefficients of the cubic regression of the ordered differences *y*_*t*_ − *y*_*t*−1_ on the observed values.

For each data set we used the synthetic likelihood Markov chain Monte Carlo (MCMC) method with 30000 iterations, a burn in of 3 time steps and 500 simulations to compute each ${\hat{\mu }}_{\theta }$ and ${\hat{\Sigma }}_{\theta }$ [26]. We used the following prior:

$$\begin{array}{c} \begin{array}{cc} r & ∼U(1,90)\\ \sigma & ∼U(0.05,0.7)\\ \phi & ∼U(0,20). \end{array} \end{array}$$ (5)

The synlik package generates the MCMC chain on a logarithmic scale, we estimated the parameters as the exponential of the posterior mean. To ensure convergence, only the last half of the chain is used (the last 15000 iterations).

#### Creation of the prepaid grid

For the prepaid estimation, we used the same summary statistics as for the traditional synthetic likelihood, except for two differences. First, the coefficients of the cubic regression of the ordered differences *y*_*t*_ − *y*_*t*−1_ on the observed values could not be used, because the observed values are not available when creating the prepaid grid. Second, we changed the number of zeros to the percentage of zeros to make this statistic independent of *T*_obs_ (as this may change depending on the observation).

We filled the prepaid grid with 100000 parameter sets using the priors of Eq 5. To cover this grid as evenly as possible (and avoiding too large gaps), the uniform distribution was approximated using Halton sequences [27, 28]. For each parameter set in the prepaid grid, we simulated a time series of length 10^7^ and used the summary statistics of this long time series as ${\hat{\mu }}_{\theta }$.

Each time series was then split into series of length *T*_*prepaid*_ = 100, 1000 and 10000 which were used to compute the covariance ${\hat{\Sigma }}_{\theta ,T_{prepraid}}$ for the statistics computed on data of these lengths. This means, for example, that we had 100000 series of length 100 to compute the covariance matrix for a certain parameter set for time series of length 100. If we need to estimate parameters of a time series with *T*_obs_ not equal to one of the *T*_prepaid_ lengths, we use the covariance matrix created with time series of length *T*_*prepaid*_ which is closest to *T*_obs_ in logarithmic scale and adapt the covariance matrix into

$$\begin{array}{c} {\hat{\Sigma }}_{\theta ,T_{\text{obs}}}=\frac{T_{\text{prepaid}}}{T_{\text{obs}}}{\hat{\Sigma }}_{\theta ,T_{\text{prepaid}}} \end{array}$$ (6)

The creation of the prepaid grid took approximately one day on a 3.4GHz 20-core processor.

To allow the estimation for a larger range of parameters for the online estimation at http://www.prepaidestimation.org/ we created a new and bigger prepaid grid using the following priors:

$$\begin{array}{c} \begin{array}{cc} \text{log}(r) & ∼U(\text{log}(1),log(200))\\ \sigma & ∼U(0.05,0.7)\\ \text{log}(\phi ) & ∼U(-2,7). \end{array} \end{array}$$ (7)

We filled to prepaid grid with 100000 parameter sets and used this prior for the real life data set on the Chilo partellus.

#### Prepaid estimation

Four variants of prepaid estimation were implemented for this example. All use the negative synthetic likelihood as distance (*d*(***s***^sim^, ***s***^obs^) as defined in the main text and Fig 1). First, we do a nearest neighbor estimation ${\text{SL}}_{\text{ML}}^{\text{GRID}}$, without using any interpolation between the grid points of the prepaid data set. We compute the synthetic likelihood of all the prepaid parameters for the summary statistics of the test data set. The parameter vector with the highest likelihood, the so-called nearest neighbor may already be a good estimation. For a low number of time points *T*_obs_, it is to be expected that the error on the parameter estimate is much larger than the gaps in the prepaid grid, and in such a case, the ${\text{SL}}_{\text{ML}}^{\text{GRID}}$ estimation approach suffices.

Second, a more accurate estimation can be acquired by interpolating between the parameter values in the prepaid grid (${\text{SL}}_{\text{ML}}^{\text{SVM}}$). Therefore, we learn the relation between the parameters and the summary statistics: ${\hat{f}}_{svm}:\theta \mapsto s$. However, we only learn this relation in the region of interest, that is the 100 nearest neighbors according to the synthetic likelihood. For each summary statistic, we create, on the fly, a separate least squares support vector machine (LS-SVM) [12] using the 100 nearest neighbors. This machine learning technique is chosen as it is a fast non-linear method which generalizes well. We limit the predictions to the possible range of the summary statistics (e.g., to prevent a percentage of zeros, one of the statistics, larger than 1).

We then use the differential evolution global optimizer [14] to find the maximum of:

$$\begin{array}{c} l_{s}^{\text{PP}}\left(\theta \right)=-\frac{1}{2}{\left(s^{\text{obs}}-{\hat{f}}_{svm}(\theta )\right)}^{T}{\hat{\Sigma }}_{\theta ,T_{obs}}^{-1}(s^{\text{obs}}-{\hat{f}}_{svm}(\theta ))-\frac{1}{2}\log|{\hat{\Sigma }}_{\theta ,T_{obs}}|, \end{array}$$ (8)

where ${\hat{\Sigma }}_{\theta ,T_{obs}}$ is the covariance matrix of the statistics of the nearest neighbor as defined in Eq 6. The superscript “PP” is used to denote that we use the prepaid version of synthetic likelihood, and not the traditional version as used by [2] (see Eq 4). The optimization process is constrained and we use the minima and maxima for each parameter of the 100 nearest neighbors as effective bounds.

The ${\text{SL}}_{\text{ML}}^{\text{SVM}}$ approach makes use of a non-linear black box interpolator. However, we may also consider using a much faster linear regression (see also the toy example in Section). Therefore, we will also compare the ${\text{SL}}_{\text{ML}}^{\text{SVM}}$ (and ${\text{SL}}_{\text{ML}}^{\text{Grid}}$) approach to a third option where we predict the summary statistics using a linear regression (called the ${\text{SL}}_{\text{ML}}^{\text{Lin}}$ approach).

Third, we can easily implement a prior for the likelihood in Eq 4. This leads to a posterior given by

$$\begin{array}{c} p\left(\theta |s^{\text{obs}}\right)\propto p\left(\theta \right)l_{s}\left(\theta \right). \end{array}$$ (9)

The parameters will be estimated as the maximum a posteriori (MAP), as comparison to maximum likelihood estimation which is a maximum a posteriori with a uniform prior. Here we will apply this extension to the nearest neighbor estimation: ${\text{SL}}_{\text{MAP}}^{\text{GRID}}$.

Lastly we will show that our prepaid method can also be used to cover an experimental set-up. In such a set-up, we want to estimate the same model over several experimental conditions. For example, we may be interested in the effect of light intensity on the population dynamics of a certain type of bacteria. In such an example we would vary the light intensity over several conditions and estimate the population dynamics again for each condition.

If, for this experimental set-up, the conditions *c* are independent, the likelihood of the whole experiment is

$$\begin{array}{c} l_{s,experiment}\left(\theta_{1},\theta_{2},...,\theta_{C}\right)=\prod_{c=1}^{C}l_{s,c}\left(\theta_{c}\right) \end{array}$$ (10)

where *l*_*s*,*c*_(***θ***_*c*_) is the synthetic likelihood for condition *c*. This is equivalent to estimating each parameter set ***θ***_*c*_ individually for each condition *c* poses no problem for the previously proposed prepaid method.

In many experimental set-ups, the conditions will however not be independent. In the case of our example, we may only be interested in the effect of light intensity on the scaling parameter *ϕ*, and expect the other parameters *r* and *σ* to be constant across conditions. Such a dependence between conditions can be mimicked using priors. In case of the experiment example, with two conditions, we propose the following prior:

$$\begin{array}{c} p\left(\theta_{1},\theta_{2}\right)=N(\frac{r_{1}-\bar{r}}{\sigma_{prior}})N(\frac{r_{2}-\bar{r}}{\sigma_{prior}})N(\frac{\sigma_{1}-\bar{\sigma }}{\sigma_{prior}})N(\frac{\sigma_{2}-\bar{\sigma }}{\sigma_{prior}}) \end{array}$$ (11)

where N is the standard normal distribution and $\bar{r}$ and $\bar{\sigma_{1}}$ are the averages of respectively *r* and *σ* across conditions ($\bar{r}=\frac{r_{1}+r_{2}}{2}$ and $\bar{\sigma }=\frac{\sigma_{1}+\sigma_{2}}{2}$). Using such a prior we can force *r*_1_ and *σ*_1_ to be similar to *r*_2_ and *σ*_2_ respectively. The smaller the tuning parameter *σ*_*prior*_, the more all constrained parameters (*r* and *σ*) will be forced to be equal. If *σ*_*prior*_ is too large the estimation will not take into account the interdependence between the conditions. So at first, it seems that *σ*_*prior*_ needs to be as small as possible. However, if *σ*_*prior*_ is too small we run into trouble with the sparsity of the prepaid grid. In the limit, where *σ*_*prior*_ goes to zero, the estimation process will choose a parameter where *r*_1_ = *r*_2_ and *σ*_1_ = *σ*_2_ will hold exactly. Due to the nature of the prepaid grid, this will lead to the undesired result where exactly one prepaid point is chosen for both conditions, meaning that also *ϕ*_1_ = *ϕ*_2_. Luckily, *σ*_*prior*_ can be easily tuned. Once the prepaid grid is created, we can estimate many test parameters using the the experimental set-up in combination with a certain tuning parameter. Subsequently, the tuning parameter which leads to the best estimates of these test parameters is chosen.

In practice, when *σ*_*prior*_ is tuned, we will first create a pool of eligible parameters for each condition individually using the nearest neighbor approach ${\text{SL}}_{\text{ML}}^{\text{GRID}}$. In a second step we fill refine these pools by using the prior of Eq 11 and choose the best estimate for each condition. In a last step we replace *r*_1_ and *r*_2_ by $\bar{r}$ and *σ*_1_ and *σ*_2_ by $\bar{\sigma }$ to ensure that the constraints of the experimental set up are exactly satisfied.

More generally, for an experiment with several conditions where we want parameter *θ* to be constant over the conditions we get the following prior:

$$\begin{array}{c} p\left(\theta_{1},\theta_{2},...,\theta_{3}\right)=\prod_{c=1}^{C}N(\frac{\theta_{c}-\bar{\theta }}{\sigma_{prior}}) \end{array}$$ (12)

#### Test set

As a test set we first used 100 random parameters created with the prior of Eq 5. To avoid problems with the borders we deleted parameters that where within 1% range of the bounds. We simulated data sets for *T*_obs_ = {10^2^, 5⋅10^2^, 10^3^, 10^4^, 10^5^}. For each data set we estimated parameters using the nearest neighbor (${\text{SL}}_{\text{ML}}^{\text{Grid}}$) and the ${\text{SL}}_{\text{ML}}^{\text{SVM}}$ approach. For *T*_obs_ = 10^5^, we also estimated the parameters using the ${\text{SL}}_{\text{ML}}^{\text{Lin}}$ approach. Due to time constraints, we only estimated parameters for the data with *T*_obs_ ≤ 10^3^ using the traditional synthetic likelihood approach.

Next we also created test data sets from different priors for *T*_obs_ = 10^2^. Prior *P*_1_ from Eq 5 can also be written as

$$\begin{array}{c} \begin{array}{cc} \frac{r-1}{90-1} & ∼Beta(1,1)\\ \frac{\sigma -0.05}{0.7-0.05} & ∼Beta(1,1)\\ \frac{\phi }{20} & ∼Beta(1,1). \end{array} \end{array}$$ (13)

where *Beta* is a beta distribution with parameters *α* = 1 and *β* = 1. Similarly, we created a test set from prior *P*_2_

$$\begin{array}{c} \begin{array}{cc} \frac{r-1}{90-1} & ∼B(10,10)\\ \frac{\sigma -0.05}{0.7-0.05} & ∼B(10,10)\\ \frac{\phi }{20} & ∼B(10,10), \end{array} \end{array}$$ (14)

and prior *P*_3_

$$\begin{array}{c} \begin{array}{cc} \frac{r-1}{90-1} & ∼B(2,10)\\ \frac{\sigma -0.05}{0.7-0.05} & ∼B(10,2)\\ \frac{\phi }{20} & ∼B(2,10). \end{array} \end{array}$$ (15)

We will test if ${\text{SL}}_{\text{MAP}}^{\text{GRID}}$ performs best when the correct prior is used in the estimation process. Last we also created a test set for *T*_obs_ = 10^2^ for an experimental set up with two conditions where *r* and *σ* are equal over the conditions.

In the subsequent sections, we will evaluate the methods on the following criteria: accuracy, speed and coverage.

#### Results accuracy

**Fig 6:**
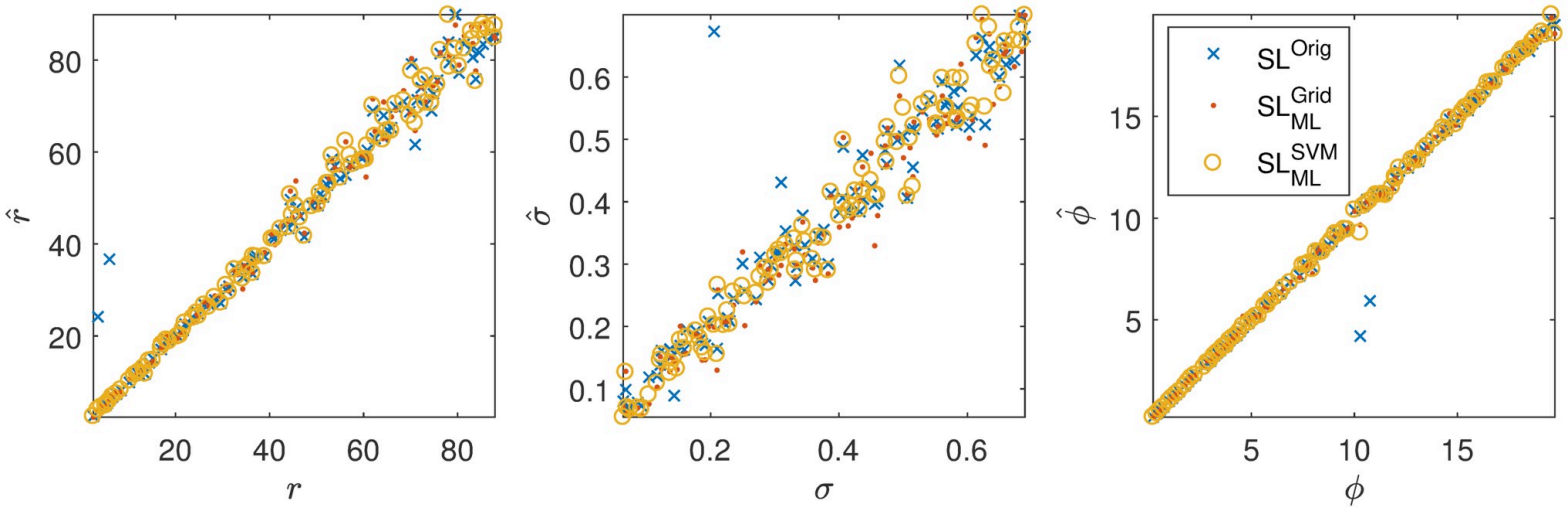
Estimated versus true parameters of the Ricker model of 100 data sets with *T*_obs_ = 1000. The SL^Orig^ estimation has some problems with outliers.

To start off, we look at the recoveries for *T*_obs_ = 10^3^ for all 100 simulated data sets and the three methods (SL^Orig^,${\text{SL}}_{\text{ML}}^{\text{Grid}}$ and ${\text{SL}}_{\text{ML}}^{\text{SVM}}$). Scatter plots are shown in Fig 6. It can seen that the synthetic likelihood estimation leads to some clear outliers. One possible reason for the absence of outliers in the prepaid estimation is the fact that prepaid estimation from the start examines the whole grid and therefore has less problems with getting stuck in local optima.

**Fig 7:**
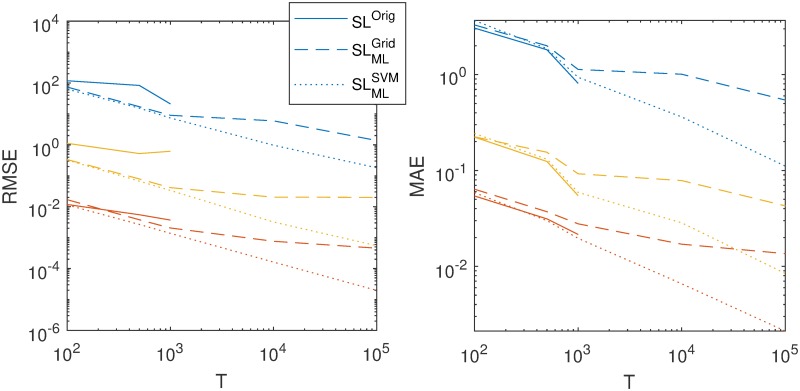
The accuracy of all estimation methods versus the number of time points *T*_obs_. The left panel shows the mean squared error, while the right panel shows the median absolute error. The three colors represent the three parameters. Blue lines refer to the parameter *r*, red lines to the parameter *σ* and yellow lines to the parameter *ϕ*. The solid line represents the original synthetic likelihood approach SL^Orig^ (stopping at *T*_obs_ = 10^3^), the dashed line the ${\text{SL}}_{\text{ML}}^{\text{SVM}}$ prepaid approach and the dotted line the ${\text{SL}}_{\text{ML}}^{\text{SVM}}$ prepaid approach.

More generally, we plotted the accuracy of each of the methods as a function of time series length *T*_obs_ in Fig 7. The left panel shows the root mean square error (RMSE), while the right panel shows the median absolute error (MAE). We decided to look at the MAE because the few outliers for SL^Orig^ (which were shown Fig 6) may inflate the RMSE of the synthetic likelihood disproportionally, which happens to a certain extent. However, very similar conclusions can be drawn for both performance measures. In general, accuracy increases when *T*_obs_ increases (i.e., both RMSE and MAE decreases). For RMSE, our SVM prepaid method clearly outperforms the traditional synthetic likelihood method SL^Orig^ for every *T*_obs_ and every parameter. For *T*_obs_ = {5⋅10^2^, 10^3^}, also the ${\text{SL}}_{\text{ML}}^{\text{Grid}}$ prepaid approach leads for every parameter to a lower RMSE compared to the synthetic likelihood. For all *T*_obs_, the ${\text{SL}}_{\text{ML}}^{\text{SVM}}$ prepaid leads to a higher accuracy compared to the ${\text{SL}}_{\text{ML}}^{\text{Grid}}$ prepaid and this difference becomes larger for a larger *T*_obs_. For MAE, the ${\text{SL}}_{\text{ML}}^{\text{SVM}}$ prepaid method and the original synthetic likelihood SL^Orig^ show a very similar accuracy (for *T*_obs_ ≤ 10^3^). Both outperform the ${\text{SL}}_{\text{ML}}^{\text{Grid}}$ prepaid.

The largest attainable accuracy for the ${\text{SL}}_{\text{ML}}^{\text{Grid}}$ prepaid approach is limited by the spacing of the prepaid grid. If we had created an equally spaced grid of *T*_obs_ = 10^5^ points using the prior in Eq 5, we would have the following gaps in each of the three parameter dimensions:

$$\begin{array}{c} \begin{array}{cc} \Delta_{r} & =\frac{90-1}{{\left(10^{5}\right)}^{1/3}}=1.9\\ \Delta_{\sigma } & =\frac{0.7-0.05}{{\left(10^{5}\right)}^{1/3}}=0.01\\ \Delta_{\phi } & =\frac{20-0}{{\left(10^{5}\right)}^{1/3}}=0.4. \end{array} \end{array}$$ (16)

**Fig 8:**
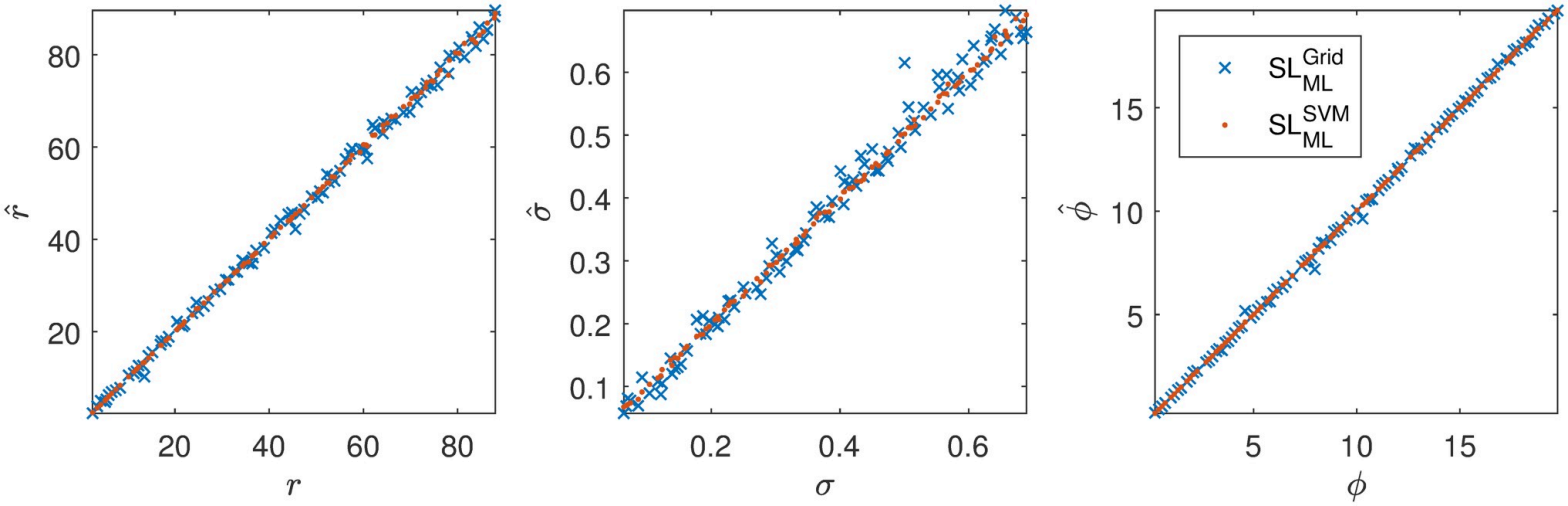
The estimation of the three parameters of the Ricker model of 100 data sets with *T*_obs_ = 10^5^. The ${\text{SL}}_{\text{ML}}^{\text{SVM}}$ estimation clearly outperforms the ${\text{SL}}_{\text{ML}}^{\text{Grid}}$ estimation.

We do not have an equally spaced grid, but it is expected that the quasi Monte Carlo distribution of points creates expected gaps close to the ones in Eq 16. Therefore, it is no coincidence that the best possible RMSE using the ${\text{SL}}_{\text{ML}}^{\text{Grid}}$ prepaid approach has the same order of magnitude as the gap size Δ, as can be seen in Table 2 for the case of *T*_obs_ = 10^5^. However, Table 2 also show that the ${\text{SL}}_{\text{ML}}^{\text{SVM}}$ prepaid approach leads to a much lower RMSE. The difference between the ${\text{SL}}_{\text{ML}}^{\text{Grid}}$ and the ${\text{SL}}_{\text{ML}}^{\text{SVM}}$ prepaid approach for *T*_obs_ = 10^5^ is further visualized in Fig 8.

**Table 2:** RMSE for the estimation of the parameters of the Ricker model for *T* = 10^5^ using the ${\text{SL}}_{\text{ML}}^{\text{Grid}}$, ${\text{SL}}_{\text{ML}}^{\text{SVM}}$ and ${\text{SL}}_{\text{ML}}^{\text{Lin}}$ prepaid methods.

	r	*σ*	*ϕ*
${\text{SL}}_{\text{ML}}^{\text{Grid}}$	1.2	0.021	0.14
${\text{SL}}_{\text{ML}}^{\text{SVM}}$	0.43	0.0044	0.023
${\text{SL}}_{\text{ML}}^{\text{Lin}}$	0.54	0.013	0.091

The results in Table 2 also show the need for a non-linear interpolator for the prepaid method. The RMSE of a linear regression interpolator (${\text{SL}}_{\text{ML}}^{\text{Lin}}$) is much larger than that of the SVM prepaid.

In sum, we can conclude that the prepaid estimation methods lead to better, or at least similar, results as the traditional synthetic likelihood.

#### Results speed

The largest improvement of the prepaid method over synthetic likelihood is in computational speed: The prepaid method is many times faster than synthetic likelihood. Consider Fig 2 in the main text where it is shown that the ${\text{SL}}_{\text{ML}}^{\text{Grid}}$ prepaid method is finished before a single iteration of the 30000 iterations are done by the SL^Orig^ method. While the ${\text{SL}}_{\text{ML}}^{\text{Grid}}$ and the ${\text{SL}}_{\text{ML}}^{\text{SVM}}$ prepaid methods are finished in respectively 0.044 and 3.7 seconds, independent of the time series length *T*_obs_, the SL^Orig^ method grows slower with an order of magnitude of *T*_obs_. In each SL^Orig^ iteration one needs to simulate multiple time series with length *T*_obs_. The larger *T*_obs_, the slower the estimation. While the synthetic likelihood needs approximately one and a half hour to estimate the parameters for a time series with length *T*_obs_ = 10^3^. The ${\text{SL}}_{\text{ML}}^{\text{Grid}}$ prepaid estimation still finishes in 0.044 s, which is more than 10^5^ times faster. The speed up factors are presented in Table 3 and as can be seen from Fig 7, there is not loss of accuracy. The speed up would reach millions, if we had the time to run the synthetic likelihood method for longer time series.

**Table 3:** Average time in seconds needed for the SL^Orig^ estimation for multiple *T*_obs_ and the speed up for the ${\text{SL}}_{\text{ML}}^{\text{Grid}}$ and ${\text{SL}}_{\text{ML}}^{\text{SVM}}$ methods. The time for *T*_obs_ = 10^4^ and *T*_obs_ = 10^5^ was not measured, so these values are estimated and between brackets. (Fig 7 shows the corresponding accuracies).

*T*_obs_	10^2^	5 ⋅ 10^2^	10^3^	10^4^	10^5^
time SL^Orig^	716 s	3549 s	5841 s	(50000 s)	(500000 s)
${\text{SL}}_{\text{ML}}^{\text{GRID}}$ times faster	16273	80659	132750	(1000000)	(10000000)
${\text{SL}}_{\text{ML}}^{\text{SVM}}$ times faster	194	959	1578	(10000)	(100000)

#### Results coverage

Next, we look at the coverage rates of the 95% confidence intervals as obtained with the bootstrap in combination with the prepaid method. To estimate a 95% confidence interval of the estimates for the prepaid method, a parametric bootstrap with *B* = 1000 bootstrap samples was used.

For the prepaid version the estimate for the observed data set was obtained using the ${\text{SL}}_{\text{ML}}^{\text{SVM}}$ approach and the bootstrap estimates were commonly obtained using the ${\text{SL}}_{\text{ML}}^{\text{Grid}}$ prepaid method applied to the bootstrap data sets. However, if in the first 100 bootstraps only half of the nearest neighbors where unique points, the bootstrap distribution could be considered questionable. This behavior is to be expected for larger sample sizes *T*_obs_, because the true bootstrap distribution is very peaked so that every bootstrap sample will have the same nearest neighbor grid point. When this occurs, we would estimate the parameters of each bootstrap using differential evolution, using the SVM created by the original 100 nearest neighbors.

Alternatively, for the synthetic likelihood approach (using MCMC) we computed the 95% confidence interval by calculating the 0.025 and 0.975 quantiles of the last half of the posterior samples.

The coverage results for the test set of 100 parameters are shown for three different values of *T*_obs_ in Table 4. It can be seen that for both methods, the coverage is close to the nominal level of 95%, but the coverage of the prepaid method is slightly better.

**Table 4:** The effective coverages of the test set for different *T*_obs_.

	*T*_obs_	r	*σ*	*ϕ*
SL^Orig^	10^2^	0.9	0.89	0.93
	5 ⋅ 10^2^	0.94	0.92	0.94
	10^3^	0.92	0.91	0.92
prepaid	10^2^	0.95	0.84	0.97
	5 ⋅ 10^2^	0.96	0.94	0.96
	10^3^	0.97	0.95	0.97

#### Results prior

In this paragraph we show how we can benefit from using the correct prior. We estimate the parameters of the three testsets for *T*_obs_ = 100, created with uniform prior *P*_1_ from Eq 9 and beta distribution priors *P*_2_ and *P*_3_ from Eqs 14 and 15. We estimated all three data sets using maximum a posteriori estimation ${\text{SL}}_{\text{MAP}}^{\text{GRID}}$ using all three priors. The results are shown in Table 5. Using the correct prior leads, as expected, to the best results.

**Table 5:** RMSE of ${\text{SL}}_{\text{MAP}}^{\text{GRID}}$ estimation of test sets with *T*_obs_ = 100 created with priors *P*_1_, *P*_2_ and *P*_3_ and estimated by using priors *P*_1_, *P*_2_ and *P*_3_. For each test set and parameter the best result is shown in bold.

	estimated with *P*_1_			estimated with *P*_2_			estimated with *P*_3_		
parameter	r	*σ*	*ϕ*	r	*σ*	*ϕ*	r	*σ*	*ϕ*
test set created with *P*_1_	**8.2**	**0.13**	**0.53**	10	0.12	0.82	16	0.17	0.94
test set created with *P*_1_	10	0.13	0.55	**6.5**	**0.072**	**0.43**	11	0.12	0.60
test set created with *P*_1_	4.4	0.15	0.33	6.9	0.19	0.51	**3.5**	**0.065**	**0.28**

#### Parameter constraints across conditions

We estimated the parameters for a two condition experimental set up with equal *r* and *σ*, with and without the prior from Eq 11 (parameter *σ*_*prior*_ was tuned on 100 similar simulated data sets). The results are shown in Table 6. Using the prior from Eq 11, which implements the parameter constraints of the experimental set up, leads, as expected, to better results for each parameter. Even for *ϕ*, which is absent in the prior, we find better results.

**Table 6:** RMSE for Ricker model data where *T*_obs_ = 100 for an experimental set up with two conditions where *r* and *σ* are equal over the conditions. Parameters are estimated by using ${\text{SL}}_{\text{MAP}}^{\text{GRID}}$ with a flat prior (same as ${\text{SL}}_{\text{ML}}^{\text{GRID}}$)and with a prior from Eq 11.

prior	r	*σ*	*ϕ*
flat prior	88	0.17	0.42
prior Eq 11	61	0.11	0.36

#### Results real life data set

The results for the estimation of the population dynamics of the Chilo partellus [16, 15], using the prior from Eq 7 can be found in Table 7. For the prepaid, we estimated the parameters using the methods online at http://www.prepaidestimation.org/. All estimations are similar and have overlapping confidence intervals. The prepaid estimation is however significantly faster.

**Table 7:** Population dynamics of the Chilo partellus [16, 15]. We show the estimates, the 95% confidence intervals and computation time of the prepaid and synthetic likelihood estimation techniques.

	r	*σ*	*ϕ*	Time (in seconds)
SL^Orig^	1.05 (1.01– 1.1)	0.41 (0.31–0.51)	248.17 (139.53–493.2)	830
${\text{SL}}_{\text{ML}}^{\text{GRID}}$	1.10 (1.06– 1.34)	0.43 (0.30–0.54)	140.60 (43.94–208.19)	0.2
${\text{SL}}_{\text{ML}}^{\text{SVM}}$	1.06 (1.01– 1.24)	0.41 (0.21–0.56)	176.15 (19.27–427.65)	4

### Application 2: A stochastic model of community dynamics

A second model we will apply our prepaid modeling technique to, is a stochastic dispersal-limited trait-based model of community dynamics [17]. The data that will be modeled, are the abundances of species (hence a vector of frequencies, in which each component is a different species). Each species in the local environment is assumed to have a competitive value dependent on its trait *u*, given by the filtering function

$$\begin{array}{c} F\left(u\right)=1+Ae^{-\frac{{\left(u-h\right)}^{2}}{2\sigma^{2}}}. \end{array}$$ (17)

Here *A* is the maximal competitive advantage, *h* is the optimal trait value in the local environment and *σ* describes the width of the filtering function. At each time step, one individual from the local community dies. It is then replaced with a probability $1-\frac{I}{I+J+1}$ by a random descendant from the local pool. Here, *J* is the size of the local community and *I* is the fourth parameter to estimate, related to the amount of immigration from the regional pool into the local community. The probability that this descendant comes from a certain individual in the local community, is proportional to the competitiveness of this individual. With a probability of $\frac{I}{I+J+1}$, the dead individual is replaced by an immigrant from the regional pool. The distribution of traits *u* of the individuals in the regional pool is assumed to be uniform over *u*. It is noteworthy that Jabot saw the necessity of reusing ABC simulations to reduce computation time in his recovery study [17].

The model was simulated using the C++ code from the Easy ABC package [22] where a regional pool of *S* = 1000 species was defined evenly spaced on the trait axis (i.e., the resolution) and *J* = 500 was the size of the local community.

#### ABC estimation

We compare our prepaid method estimation with the Easy ABC package (ABC^Orig^) [29, 22]. Because we work in a Bayesian framework, we first have to specify priors. As in Jabot et al. we use the following priors [22]:

$$\begin{array}{c} \begin{array}{cc} \log(I) & ∼U(3,5)\\ \log(A) & ∼U(\log(0.1),\log(5))\\ h & ∼U(-25,125)\\ \log(\sigma ) & ∼U(\log(0.5),\log(25)). \end{array} \end{array}$$ (18)

In this application, the parameter vector ***θ*** is defined as follows: ***θ*** = (log(*I*), log(*A*), *h*, log(*σ*)). To get the ABC algorithm to work, we compute four summary statistics: the richness of the community (number of living species), Shannon’s index which measures the entropy of the community, and the mean and the skewness of the trait distribution of the community.

The ABC algorithm we use applies a sequential parameter sampling scheme [30]. The sequence of tolerance bounds is given by *ρ* = {8, 5, 3, 1, 0.5, 0.2, 0.1} and the algorithm proceeds to the next tolerance after 200 simulations which lead to summary statistics within the bounds. The last 200 simulations within the bounds represent the posterior, and the estimate of the parameter is given by the posterior mean.

#### Creation of the prepaid grid

For the prepaid estimation, we used exactly the same summary statistics as the Easy ABC package. We filled the prepaid grid with 500, 000 parameter vectors using the priors of Eq 18, but for most examples we will use a grid with only 100, 000 parameter vectors. To cover this grid as evenly as possible, the uniform distribution was approximated using Halton sequences [27, 28] (in order to avoid gaps that may appear when Monte Carlo samples are used). The creation of the prepaid grid with 100, 000 parameter vectors took approximately 3 days on a 3.4GHz 20-core processor.

For the community dynamics models from Eqs 17 and 18, there are several ways to simulate an almost infinitely large data set to achieve stable summary statistics. The first way is to increase the number of species *S* and the size of the local pool *J*. Unfortunately some summary statistics (the richness and the entropy) are in some unknown way dependent on these parameters. As a result, the summary statistics of a simulation with *J* = 5000 cannot be used to estimate the parameters for a setting where *J* = 500. Therefore, we chose to fix the size of the local pool *J* and the number of species *S*. It is very well possible that there are summary statistics which do not have this problem, making the prepaid grid much more universal. We chose however, for the sake of comparison with the easy ABC package to keep using these parameters.

A second way to simulate data with a very large sample size is by increasing the number of time steps. By estimating the summary statistics after each time step, when one individual from the local community dies and is replaced by another individual, we create a time series of summary statistics. Averaging the summary statistics over a sufficient large number of time points will lead to stable average values of these summary statistics. In our simulations, we applied some tinning by calculating the summary statistics every time after 500 species have died (the size of the community). The reasons is that there is not enough of variation in the summary statistics computed after the death of a single species. Next, we created time series of length *T* = 100, 000 (5 ⋅ 10^7^ species will have been replaced) for the prepaid grid and used the average of these summary statistics as ${\hat{\mu }}_{\theta }$. Using this time series we also computed ${\hat{\Sigma }}_{\theta ,T_{\text{prepaid}}}$ for *T*_prepaid_ = {1, 10, 1000, 10000}. *T*_prepaid_ = 1 is of course the setting for which the original trait model is described and for which the Easy ABC algorithm is tested. Additionally we also saved 1000 samples of time series of length *T*_prepaid_ = {1, 10, 1000, 10000}.

#### Prepaid estimation

Contrary to the first application (the Ricker model), where we used a frequentist approach, for this community dynamics model we will follow a Bayesian approach. In Bayesian statistics, the focus is on the posterior distribution of the parameters *p*(***θ***|data), which is defined as follows:

$$\begin{array}{c} p(\theta |\text{data})\propto p(\text{data}|\theta )\times p(\theta ), \end{array}$$ (19)

where *p*(data|***θ***) is the likelihood and *p*(***θ***) the prior. As the likelihood, we will use the synthetic likelihood *p*(data|***θ***) ≈ *L*_*s*_(***θ***) = exp(*l*_*s*_(***θ***)), where *l*_*s*_(***θ***) is the synthetic log-likelihood as defined in Eq 4 (based on the vector of summary statistics ***s***^obs^). Because we compress the data into summary statistics, the posterior we work with is actually an approximation to the true posterior: *p*(***θ***|***s***^*obs*^) ≈ *p*(***θ***|data) (in case the summary statistics are sufficient statistics for ***θ***, the approximation sign becomes an equality sign). The distributions from Eq 18 are the priors for the parameters.

We have studied three variants of a Bayesian version of the prepaid method. These three versions will be discussed here in increasing order of complexity. We will denote the three variants as follows: ${\text{SL}}_{\text{PM}}^{\text{Grid}}$, ${\text{ABC}}_{\text{PM}}^{\text{Grid}}$, and ${\text{ABC}}_{\text{PM}}^{\text{SVM}}$.

First we will discuss ${\text{SL}}_{\text{PM}}^{\text{Grid}}$ variant. Because the priors are all uniform (and our prepaid grid is distributed following this prior), the posterior for a data set with summary statistic ***s*** at parameter ***θ***_*p*_ of the prepaid grid is proportional to

$$\begin{array}{c} p\left(\theta |s^{\text{obs}}\right)\propto L_{s}^{\text{PP}}\left(\theta \right), \end{array}$$ (20)

where $L_{s}^{\text{PP}}\left(\theta \right)$ is the prepaid synthetic likelihood (i.e., with the mean statistics computed for a very large sample and a approximate covariance matrix given by Eq 6). The posterior mean (PM), used in this variant, using prepaid synthetic likelihood can be estimated as:

$$\begin{array}{c} \hat{\theta }|s^{\text{obs}}=\frac{\sum_{p}L_{s}^{\text{PP}}\left(\theta_{p}\right)\times \theta_{p}}{\sum_{p}L_{s}^{\text{PP}}\left(\theta_{p}\right)}. \end{array}$$ (21)

**Fig 9:**
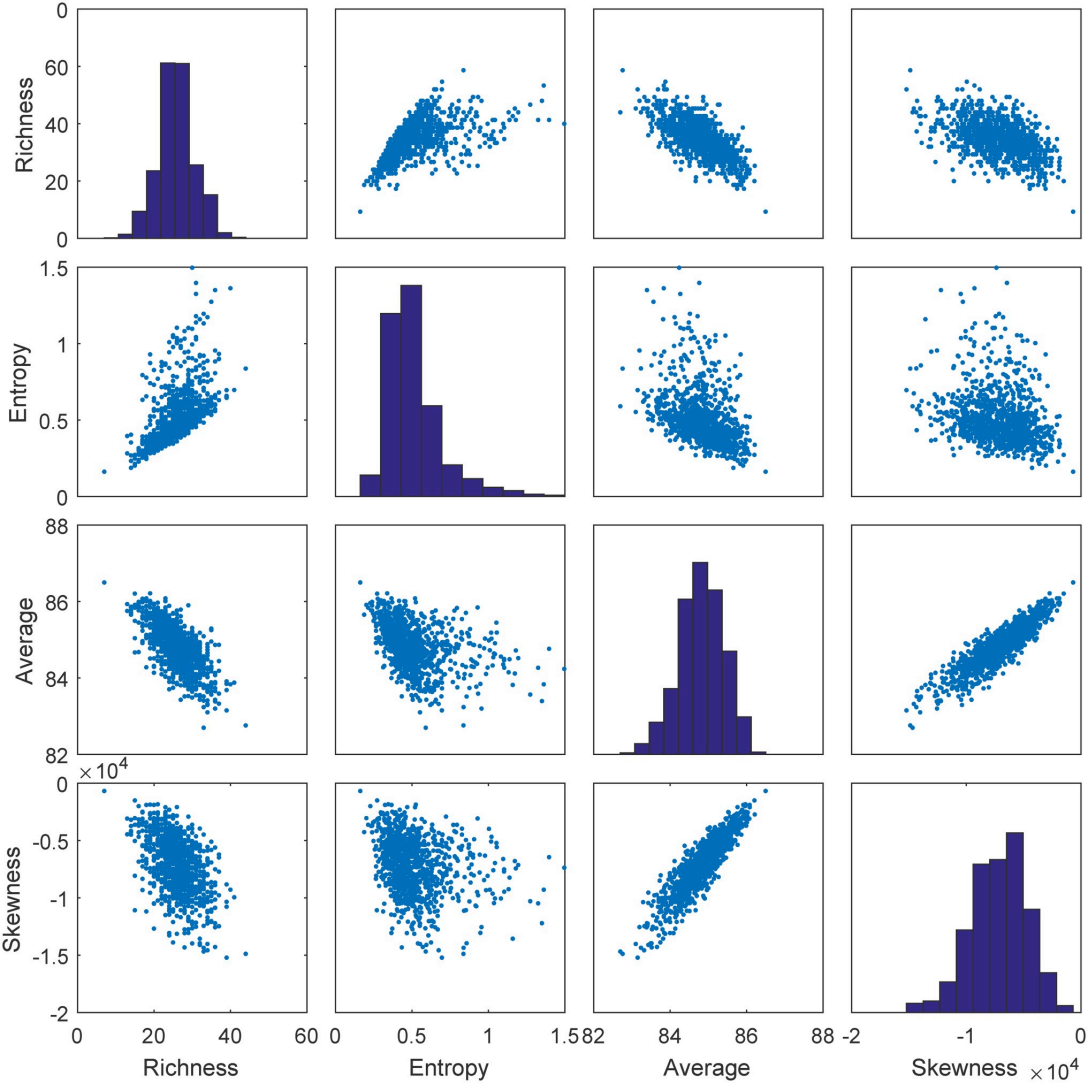
Samples for *T*_obs_ = 1 of the summary statistics of the trait model for parameter set log(*I*) = 3.0621, log(*A*) = 0.8302, *h* = 86.8924 and log(*σ*) = −0.6899.

Second we will discuss the ${\text{ABC}}_{\text{PM}}^{\text{Grid}}$ variant. The prepaid synthetic likelihood approach works best if the assumption of normally distributed summary statistics is not too far off. However, as can be seen in Fig 9, this is not always the case for the trait model defined in Eq 17. Therefore, as an alternative procedure, we propose an Approximate Bayesian Computation (ABC) approach in this variant. First, we select a subset of nearest neighbors S from the prepaid set, such that for every $\theta_{q}\in S$, the synthetic likelihood value *L*_*s*_(***θ***_*q*_) is highest and so that

$$\begin{array}{c} \frac{\sum_{q}L_{s}^{\text{PP}}\left(\theta_{q}\right)}{\sum_{p}L_{s}^{\text{PP}}\left(\theta_{p}\right)}<0.999, \end{array}$$ (22)

where the sum in the denominator runs across all grid points. In a sense, these are all the prepaid points in the 99.9% expected coverage according to the posterior of Eq 20. We denote the cardinality of S as *Q*.

In a next step, we basically perform ABC with all the grid points belonging to the selected subset S. However, there is an important issue we cannot overlook. When doing ABC, for a given parameter vector new data are simulated of the same size as the observed data. Unfortunately, our prepaid grid has correspondingly only very large data sets. To rectify this problem, so that ABC can applied without problems, we simulated during the construction of the prepaid grid, a set of *M* = 1000 prepaid samples for several designated sample sizes (i.e., *T*_prepaid_ = {1, 10, 1000, 10000}). Let us denote with $s_{q,i,T_{\text{prepaid}}}$ the vector of statistics for prepaid grid point *q*, the *i*th simulation (with *i* = 1, …, *M*) and sample size *T*_prepaid_.

Now, we can apply ABC to arrive at the posterior for ***θ***; the method will be denoted as ${\text{ABC}}_{\text{PM}}^{\text{Grid}}$. For now we will assume that *T*_obs_ is equal to one of the *T*_prepaid_ lenghts. We select the 1000 samples from this *Q* × 1000 samples set that have the smallest Mahalonobis distance to the observed set of statistics ***s***^obs^:

$$\begin{array}{c} \epsilon_{q,i,T_{\text{prepaid}}}=\left(s_{q,i,T_{\text{ABC}}}-s^{\text{obs}}\right)W_{Q}^{-1}\left(s_{q,i,T_{\text{ABC}}}-s^{\text{obs}}\right) \end{array}$$ (23)

here ***W***_*Q*_ is given by the covariance over all grid points in S and over all 1000 replications (thus, *Q* × 1000). The finally selected 1000 samples are then considered as a sample from the posterior. Note that the ${\text{ABC}}_{\text{PM}}^{\text{Grid}}$ method does not require us to progressively strengthen the tolerances, as in traditional ABC^Orig^ (governed by the tolerance parameter *ρ*). If the observed sample size *T*_obs_ is not equal to one of the *T*_prepaid_ lengths, then one can use the samples for length *T*_prepaid_ which is closest to *T*_obs_ in logaritmic scale and later adjust the posterior samples such that the posterior mean stays the same, but the posterior covariance matrix changes to

$$\begin{array}{c} {\hat{\Sigma }}_{posterior,T_{\text{obs}}}=\frac{T_{\text{prepaid}}}{T_{\text{obs}}}{\hat{\Sigma }}_{posterior,T_{\text{prepaid}}} \end{array}$$ (24)

We advise to save samples for enough different *T*_prepaid_ such that this correction is only marginal.

Lastly, we will discuss the ${\text{ABC}}_{\text{PM}}^{\text{SVM}}$ variant. The ${\text{ABC}}_{\text{PM}}^{\text{Grid}}$ is only based on the raw prepaid grid points. But again, a more accurate estimation can be found by interpolating between the parameters in the prepaid grid. Therefore, in this ${\text{ABC}}_{\text{PM}}^{\text{SVM}}$ variant, we learn the relation between the parameters and the summary statistics using LS-SVM: ${\hat{f}}_{svm}:\theta \mapsto s$. We only learn this relation in the region of interest, that is, only the 100 nearest neighbors according to the ${\text{ABC}}_{\text{PM}}^{\text{Grid}}$ approach or more specifically, the 100 prepaid points for which the most samples lead to a small enough $\epsilon_{q,i,T_{\text{ABC}}}$.

**Fig 10:**
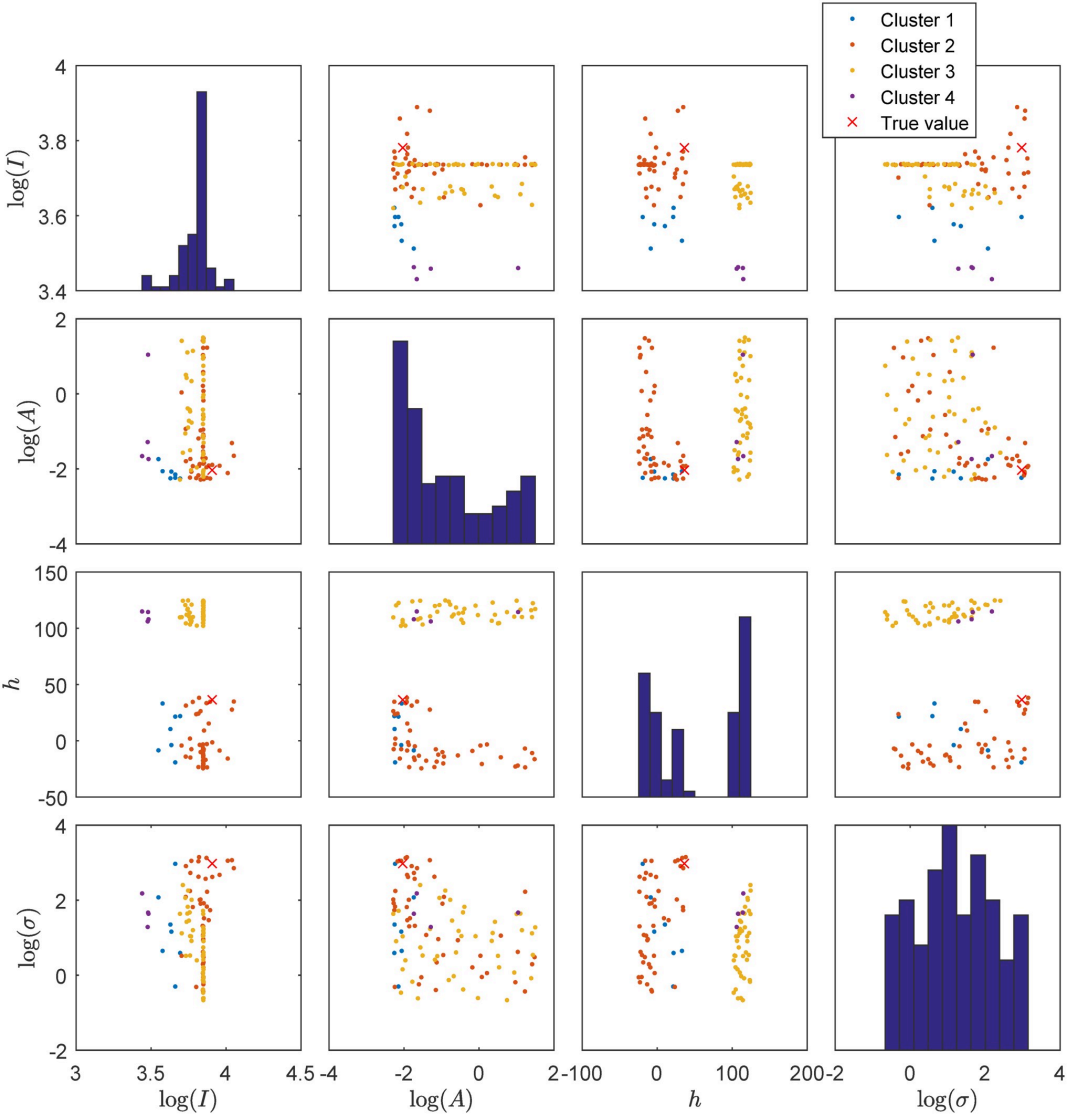
Scatter plot matrix of the clustering that occurs for the 100 nearest neighbors for the summary statistics for *T*_obs_ = 1000 of parameter log(*I*) = 3.9081, log(*A*) = −2.0343, *h* = 36.4150 and log(*σ*) = 2.9762. The red cross shows the true value of this parameter.

Before we use machine learning to infer the relation ${\hat{f}}_{svm}:\theta \mapsto s$ we cluster these 100 nearest neighbors using hierarchical clustering such that no cluster has more than 50 prepaid points. This is necessary as these 100 nearest neighbors may come from totally different areas in the prepaid grid. This is illustrated in Fig 10.

For each cluster, we first make sure that at least 20 points are included (if not, we add points from the prepaid grid which are closest). Then we estimate the ${\hat{f}}_{svm}:\theta \mapsto s$ using LS-SVM for each cluster *c* separately, giving rise to ${\hat{f}}_{svm,c}$. Next, we find the minimum volume ellipse encompassing all the points in each cluster. These ellipses inform us about the areas for which the relation holds. Subsequently we resample parameters in each ellipse to zoom in more and more to the regions of interests. In detail, we do the following in every cluster *c*:

1. Uniformly sample 1000 points ***θ***_*j*,*c*_ in the minimum volume ellipse for cluster *c*. We create a finer grid for each elipse.
2. Find the summary statistics based on the LS-SVM in cluster *c*: ${\hat{s}}_{j,c}={\hat{f}}_{svm,c}\left(\theta_{j,c}\right)$
3. Find for each point ***θ***_*j*,*c*_ the nearest point ***θ***_*p*_ from the prepaid points with which this particular cluster was created
4. Translate the 1000 samples from the nearest point ***θ***_*p*_ to the newly sampled point ***θ***_*j*,*c*_ and add to each sample the difference in summary statistics: $d={\hat{s}}_{j,c}-s_{p}$. In this step we aproximate a distribution of statistics for ***θ***_*j*,*c*_ around ${\hat{s}}_{j,c}$.
5. Keep the points ***θ***_*j*_ for which *ϵ*_*j*,*i*_ from Eq 23 is among the 5000 smallest distances and remove all others.
6. Recalculate the minimum volume ellipse with the new points.
7. Go back to step 1, until the worst *ϵ*_*j*,*i*_ does not decrease any more.

Broadly speaking, in step 1, we sample parameters ***θ***_*j*,*c*_, in step 2 to 4 we approximate the summary statistics distribution for each ***θ***_*j*,*c*_ using LS-SVM and in step 5 to 7 we trim this set of parameters to only keep the parameters with a high posterior probability.

In the end we combine all the samples, we build the posterior with the parameters from the 1000 best samples over all clusters according to Eq 23. Note that some parameters may show up several times in this posterior sample. To compute the posterior mean, we use a weighted version of these samples. The weights are given by the volume of the ellipse from the cluster where they were created. This is necessary to ensure the correct use of the equal prior for all clusters.

#### Test set

To generate the test set, we follow the same logic as in [17]. We use the prior in Eq 18 to generate 1000 random parameter sets, except for *h*, where we changed the prior with the following generating distribution:

$$\begin{array}{c} h∼U(0,100), \end{array}$$ (25)

such that 0 and 100 are the true minimum and maximum optimal trait values for communities. By taking the prior for *h* as in Eq 18, we avoid boundary effects. To exclude other problems at the borders of the parameter space, we deleted parameters which where within 1% range of the bounds. We simulated data sets for both *T*_obs_ = 1 and *T*_obs_ = 1000.

#### Results accuracy

Let us first look at the results for *T*_obs_ = 1. We have used traditional ABC (ABC^Orig^), prepaid Bayes approach based on the synthetic likelihood (${\text{SL}}_{\text{PM}}^{\text{Grid}}$) and prepaid ABC based on separately generated samples at the grid points (${\text{ABC}}_{\text{PM}}^{\text{Grid}}$ and ${\text{ABC}}_{\text{PM}}^{\text{SVM}}$). We have used 10^5^ and 5 ⋅ 10^5^ prepaid grid points. The RMSE and MAE can be found in Tables 1 and 8. All methods result in accuracies that are equally large. For 3 out of 4 parameters (except for *h*), the prepaid method outperforms ABC^Orig^ with respect to RMSE. For MAE, the prepaid method uniformly outperforms the Easy ABC package (ABC^Orig^). Overal, the difference between Ω = 10^5^ and Ω = 5 ⋅ 10^5^ prepaid grid point is very small for the prepaid methods.

**Table 8:** The MAE of the estimations of the test set of the trait model.

*T*_obs_	version	Ω	log(*I*)	log(*A*)	*h*	log(*σ*)
1	ABC^Orig^	/	0.11	0.45	1.4	0.45
1	${\text{SL}}_{\text{PM}}^{\text{Grid}}$	100000	0.1	0.39	0.96	0.38
1	${\text{ABC}}_{\text{PM}}^{\text{Grid}}$	100000	0.1	0.4	1	0.4
1	${\text{ABC}}_{\text{PM}}^{\text{Grid}}$	500000	0.1	0.38	1	0.39
1000	${\text{ABC}}_{\text{PM}}^{\text{Grid}}$	100000	0.03	0.14	0.39	0.32
1000	${\text{ABC}}_{\text{PM}}^{\text{Grid}}$	500000	0.02	0.09	0.27	0.22
1000	${\text{ABC}}_{\text{PM}}^{\text{SVM}}$	100000	0.02	0.07	0.18	0.14
1000	${\text{ABC}}_{\text{PM}}^{\text{SVM}}$	500000	0.01	0.07	0.17	0.15

We have refrained from interpolating with the LS-SVM because the 99.9% coverage includes on average more than 1000 points. This is perfectly logical because *T*_obs_ = 1 does not provide a lot of information, and, as a consequence, there is a lot of uncertainty (which translates itself into a large number of parameter points that have a reasonable large synthetic likelihood value). As a result, creating a posterior based on only 100 nearest neighbors (even after interpolation) would not suffice because too many parameter points with high posterior density would be missed.

For *T*_obs_ = 1000 (see again Tables 1 and 8), the accuracy increases, as is expected (this can be seen both in the RMSE as in the MAE). In this case, both increasing the number of grid points Ω and using LS-SVM interpolation increases accuracy. No results are given for ABC^Orig^, because it is impossible to fit the model with this sample size in acceptable time.

#### Results speed

For *T*_obs_ = 1, the estimation time of ABC^Orig^ is 3865 s. In contrast, the estimation time of ${\text{ABC}}_{\text{PM}}^{\text{Grid}}$ is 0.167 s. This means that the prepaid ABC method is approximately 23000 times faster than traditional ABC.

#### Results coverage

For both the ABC^Orig^ as well as the prepaid versions we end up with a posterior sample. We computed the coverage by calculating the 0.025 and 0.975 quantiles of the posterior samples. Next, we checked whether the true parameter was in this interval or not. Note that when we use clustering during ${\text{ABC}}_{\text{PM}}^{\text{SVM}}$, we weigh each point proportional to the volume of its originating cluster. For the ${\text{SL}}_{\text{PM}}^{\text{Grid}}$ approach we use the whole prepaid set as posterior and us weights according to Eq 20.

For *T*_obs_ = 1 and *T*_obs_ = 1000, coverage results can be found in Table 9. For *T*_obs_ = 1, ABC^Orig^ leads to better coverages than ${\text{SL}}_{\text{PM}}^{\text{Grid}}$. Also the ${\text{ABC}}_{\text{PM}}^{\text{Grid}}$ method gives good coverages (around the nominal level of 0.95) for *T*_obs_ = 1, but these coverages deteriorate for *T*_obs_ = 1000 if no interpolation is used (coverage is a bit better for 5 ⋅ 10^5^ grid points). When the LS-SVM interpolation is applied (i.e., ${\text{ABC}}_{\text{PM}}^{\text{SVM}}$), coverages become very good again, certainly for the largest number of grid points.

**Table 9:** The effective 95% coverage of the estimations of the test set of the trait model.

*T*_obs_	version	Ω	log(*I*)	log(*A*)	*h*	log(*σ*)
1	ABC^Orig^	/	0.97	0.97	0.99	0.96
1	ABC^Orig^	100000	0.84	0.87	0.86	0.86
1	${\text{ABC}}_{\text{PM}}^{\text{Grid}}$	100000	0.94	0.95	0.95	0.94
1	${\text{ABC}}_{\text{PM}}^{\text{Grid}}$	500000	0.94	0.95	0.94	0.94
1000	${\text{ABC}}_{\text{PM}}^{\text{Grid}}$	100000	0.27	0.3	0.29	0.27
1000	${\text{ABC}}_{\text{PM}}^{\text{Grid}}$	500000	0.47	0.5	0.48	0.48
1000	${\text{ABC}}_{\text{PM}}^{\text{SVM}}$	100000	0.93	0.94	0.96	0.93
1000	${\text{ABC}}_{\text{PM}}^{\text{SVM}}$	500000	0.96	0.95	0.96	0.95

### Application 3: The Leaky Competing Accumulator

Elementary decision making has been studied intensively in humans and animals [31]. A common example of an experimental paradigm is the random-motion dot task: the participant has to decide whether a collection of dots (of which only a fraction moves coherently; the others move randomly) is moving to the left or to the right. The stimuli typically have varying levels of difficulty, determined by the fraction of dots moving coherently.

Assuming there are two response options (e.g., left and right), the Leaky Competing Accumulator consists of two evidence accumulators, *x*_1_(*t*) and *x*_2_(*t*) (where *t* denotes the time), each associated with one response option. The evolution of evidence across time for a single trial is then described by the following system of two stochastic differential equations:

$$\begin{array}{c} \begin{array}{cc} dx_{1} & =\left(v+\Delta v_{i}-\gamma x_{1}-\kappa x_{2}\right)\cdot dt+c\cdot dW_{1}\\ dx_{2} & =\left(v-\Delta v_{i}-\gamma x_{2}-\kappa x_{1}\right)\cdot dt+c\cdot dW_{2}, \end{array} \end{array}$$ (26)

where *dW*_1_ and *dW*_2_ are uncorrelated white noise processes. To avoid negative values, the evidence is set to 0 whenever it becomes negative: *x*_1_ = max(*x*_1_, 0) and *x*_2_ = max(*x*_2_, 0). The initial values (at *t* = 0) are (*x*_1_, *x*_2_) = (0, 0).

The evidence accumulation process continues until one of the accumulators crosses a boundary *a* (with *a* > 0). The coordinate that crosses its decision boundary first, determines the choice that is made and the time of crossing is seen as the decision time. The observed choice response time is seen as the sum of the decision time and a non-decision time *T*_*er*_, to account for the time needed to encode the stimulus and emit the response.

Eq 26 describes the evolution of information accumulation for a two-option choice RT task, given the presentation of a single stimulus. For all stimuli, the total evidence is equal to *v*, but the differential evidence for option 1 compared to 2 is 2Δ*v*_*i*_, which is stimulus dependent and reflects the stimulus difficulty. In this example, we assume the stimuli can be categorized into four levels of difficulty, hence *i* = 1, …, 4.

The model gives rise to two separate choice response time probability densities, *p*_1*i*_(*t*) and *p*_2*i*_(*t*), each representing the response time conditional on the choice that was made. Integrating the densities over time will result in the probability of choosing the response options: $\int_{0}^{\infty }p_{1i}(t)dt=\text{Pr}(\text{option}\;1\;\text{for}\;\text{stimulus}\;i)$ and $\int_{0}^{\infty }p_{2i}(t)dt=\text{Pr}(\text{option}\;2\;\text{for}\;\text{stimulus}\;i)$. Obviously, when taken together, *p*_1*i*_ and *p*_2*i*_ sum to one.

All parameters in the parameter vector ***θ*** = (*v*, Δ*v*_1_, …, Δ*v*_4_, *κ*, *γ*, *a*, *T*_*er*_) can take values from 0 to ∞. This parametrization is known to have one redundant parameter [24], so we choose to fix *c* = 0.1.

#### The re-parametrization

The prepaid method will not be applied to the model as presented in Eq 26, but rather on a re-parametrized formulation:

$$\begin{array}{c} \begin{array}{cc} dx_{1it} & =D\cdot (v^{\prime }\left(1+C_{i}\right)-\gamma^{\prime }\cdot x_{1it}-\kappa^{\prime }\cdot x_{2it})\cdot dt+\sqrt{D}\cdot dW_{1it}\\ dx_{2it} & =D\cdot (v^{\prime }\left(1-C_{i}\right)-\gamma^{\prime }\cdot x_{2it}-\kappa^{\prime }\cdot x_{1it})\cdot dt+\sqrt{D}\cdot dW_{2it}, \end{array} \end{array}$$ (27)

again with the additional restriction that *x*_1*it*_ = max(*x*_1*it*_, 0) and *x*_2*it*_ = max(*x*_2*it*_, 0). The new parameters are defined as follows in terms of the original ones:

$$\begin{array}{ccc} D & = & c^{2}\\ v^{\prime } & = & \frac{v}{D}\\ C_{i} & = & \frac{\Delta v_{i}}{v}\\ \gamma^{\prime } & = & \frac{\gamma }{D}\\ \kappa^{\prime } & = & \frac{\kappa }{D}. \end{array}$$

This new parametrization has the advantage that *D* can be interpreted as an inverse time scalar because doubling *D* makes all choice response times twice as fast. This property will allow us to reduce the dimensionality of the prepaid grid (see below). The parameter *v*′ > 0 denotes general stimulus strength scaled according to *D*, while parameter *C*_*i*_ (for coherence) denotes the amount of relative evidence encoded in the stimulus *i*: −1 < *C*_*i*_ < 1. It is commonly assumed for these evidence accumulator models that different stimuli should lead to different coherences *C*_*i*_, but without affecting the other parameters. The nondecision time *T*_*er*_ is not transformed.

#### Creation of the prepaid grid

For the delineation of the parameter space, we will follow the specifications of [24]. Because this parameter space is rather restrictive (a consequence of the recommendation of [24] to improve parameter recovery), we will extend it through the use of a time scale parameter. This extension will be further discussed when introducing the test set.

First, we create a prepaid grid on a four-dimensional space in the original parametrization by drawing from the following distribution:

$$\begin{array}{c} \begin{array}{cc} a & ∼U(0.05,0.25)\\ v & ∼U(0.8,1.5)\\ \gamma & ∼U(1,8)\\ \kappa & ∼U(1,8). \end{array} \end{array}$$ (28)

We select 10000 grid points from this distribution using Halton sequences [27, 28]. When working in the reparametrized version, as defined in Eq 27, this space can be transformed to a four dimensional space of *v*′, *γ*′, *κ*′ and *D*.

**Fig 11:**
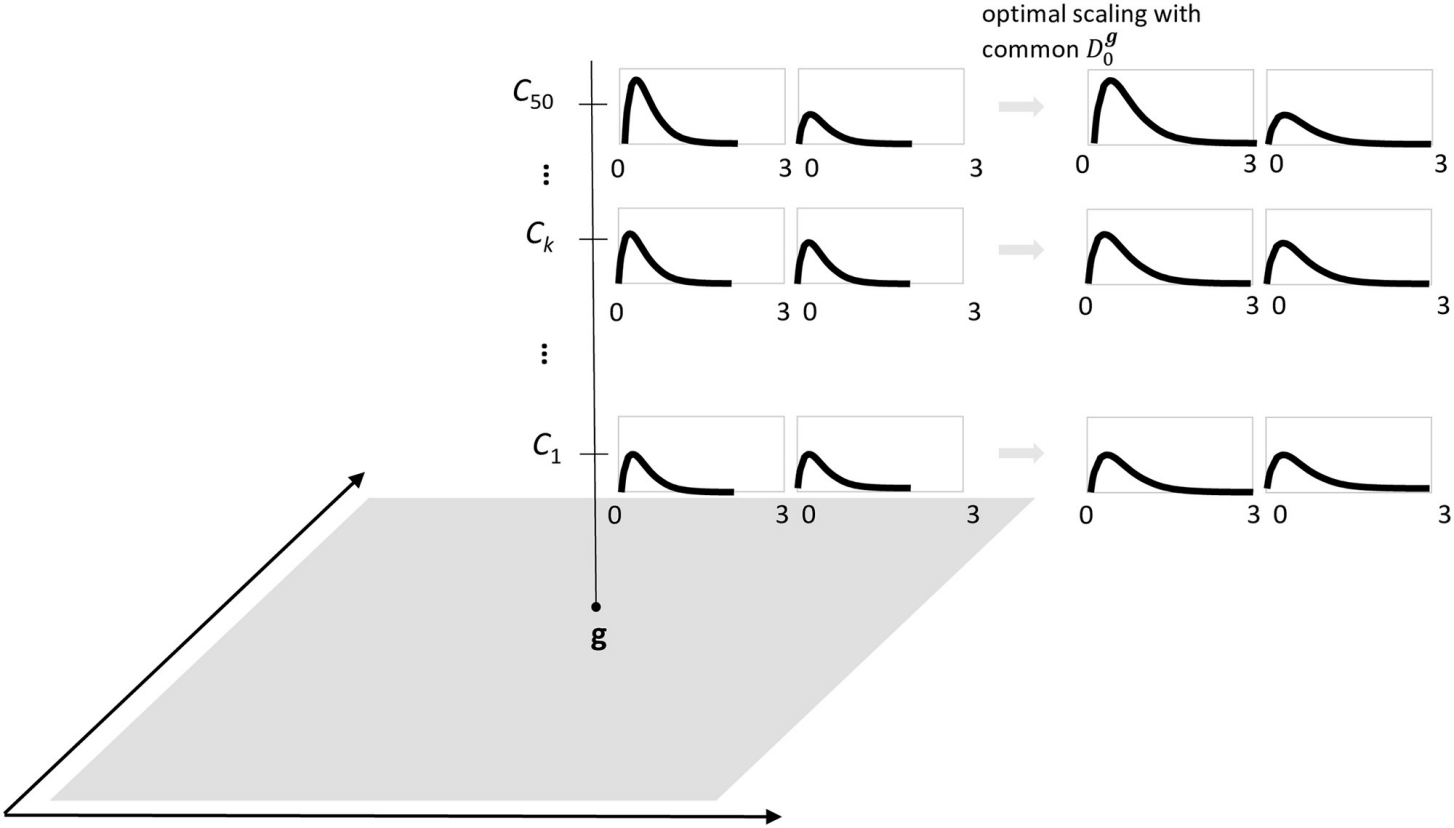
Illustration of how different coherences are incorporated. The gray plane is a simplified representation of the three dimensional (*v*′, *γ*′, *κ*′)-space. For each point ***g***, 50 coherences are chosen. Corresponding to each coherence, there is a pair of RT distributions (which each integrate to the probability of selecting the corresponding option).

**Fig 12:**
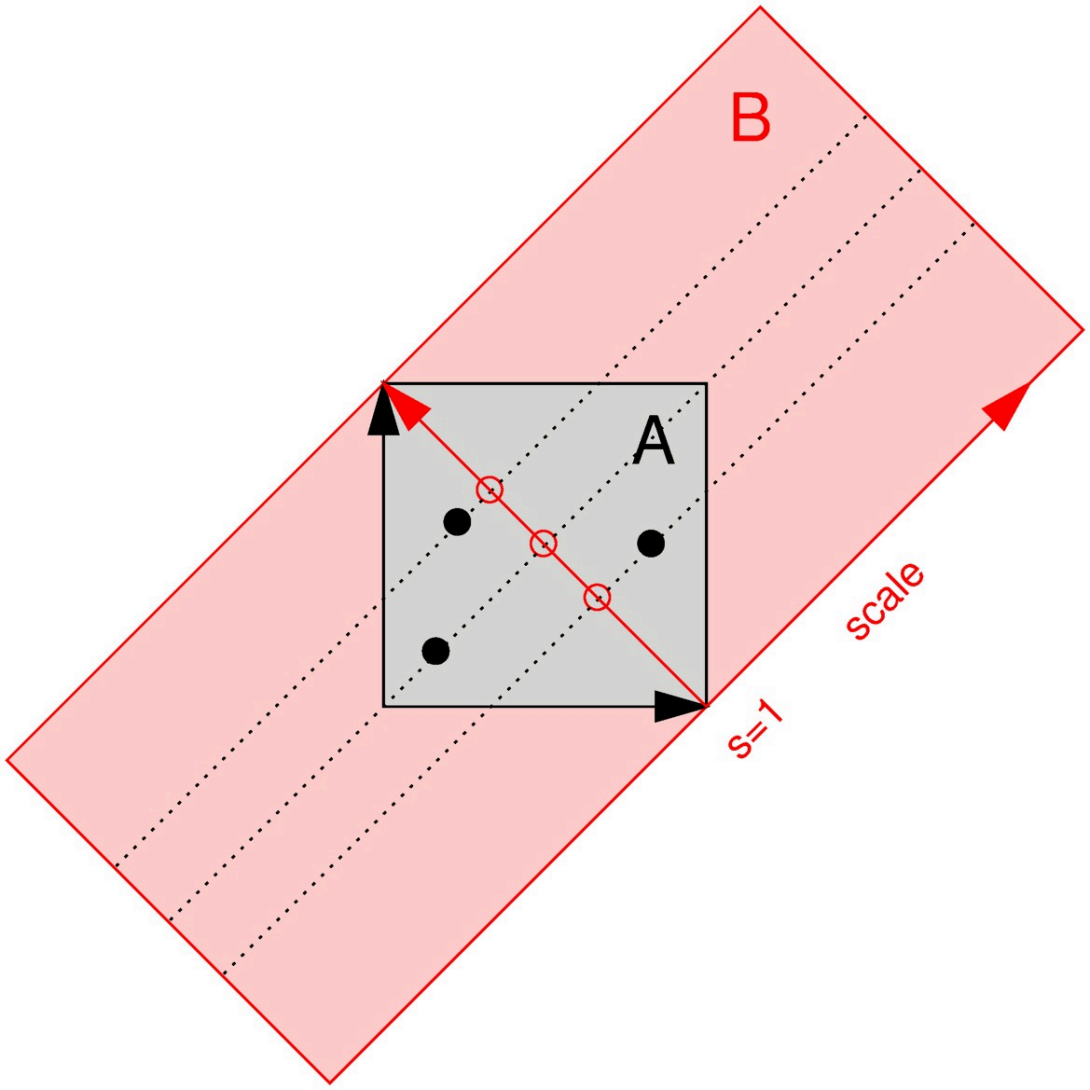
Illustration of the transformation of the original parameter space (called *A*) to a new one (called *B*) in which *D* is one of the parameters. The projections of the three parameter points on the red axis governing the width of the *B* area are denoted with open circle and these are the parameter points ***g***. For each of these open circle points, the RT distribution scales are set to 1 (i.e., *s* = 1) by choosing an appropriate diffusion coefficient (denoted as $D_{0}^{g}$) and any parameter point in *B* can be reached by selecting an appropriate ***g*** and then adjusting the scale up- or downwards (this is indicated by the dotted lines in the length direction of the new parameter space *B*.

However, because *D* acts an inverse time scalar on the response time distributions, we may also consider the three dimensional space formed by *v*′, *γ*′, and *κ*′ and for each grid point, choose the parameter *D* in such a way that the RT distributions for options 1 and 2 are scaled to fit nicely between 0 and 3 seconds (with a resolution of 1ms and 3000 time points so that about 0.0001 of the tail mass is allowed to be clipped at 3 seconds when *C* = 0). Effectively, this brings all RT distributions to the same scale (denoted as *s* = 1). This process of scaling is illustrated in Fig 11. It reduces both the number of simulations and the storage load (without it we would have to simulate and store a separate set of distributions for each value of *D*). Note that the scaling is done jointly for all RT distributions associated with a particular ***g***. The resulting diffusion constant corresponding to the rescaled distribution is denoted as $D_{0}^{g}$. In addition, the construction effectively removes one parameter from the prepaid grid, which is illustrated in Fig 12.

To include the coherence parameter, we extend each grid point with a set of predefined coherences. For each point ***g*** = (*v*′, *γ*′, *κ*′) in the grid, we take 50 equally spaced coherences $C_{k}^{g}$ (with *k* = 1, …, 50) from 0 to the maximum coherence that still has some non-zero chance of choice option 2 to be selected (we take 0.001). Finally, we simulate for each combination of ***g*** = (*v*′, *γ*′, *κ*′) and $C_{k}^{g}$ a large number of choice response time data (choices and response times). This is illustrated in Fig 11.

In a last step, grid points are eliminated from the prepaid grid, if the simulations result in too many simultaneous arrivals (i.e., trajectories that end at or very close to the intersection point of the two absorbing boundaries at the upper right corner, located at (*a*, *a*)). More specifically, we drop grid points with more than 0.1 percent simultaneous arrivals. Creating the prepaid database took less then a day on a NVIDIA GeForce GTX 780 GPU.

#### Prepaid estimation

To explain how the prepaid estimation of the LCA works, let us start with a prototypical experimental design. Assume a choice RT experiment with four stimulus difficulty levels (e.g., four coherences in the random dot motion task). Each difficulty level is administered *N* times to a single participant. A particular trial in this experiment results in (*c*_*ij*_, *t*_*ij*_), where *i* is the stimulus difficulty level (*i* = 1, …, 4) and *j* is the sequence number within its difficulty level (*j* = 1, …, *N*). The data resulting from this experiment are responses *c*_*ij*_ (referring to choice 1 or choice 2) and response times *t*_*ij*_. Each pair (*c*_*ij*_, *t*_*ij*_) is considered to originate from an unknown parameter set (*v*′, *γ*′, *κ*′, *D*, *T*_*er*_) and coherences *C*_*i*_ (*i* = 1, …, 4).

Our first aim is to is to establish a local net of prepaid points that lead to data that are close to the observed dataset. If necessary, we can further zoom in with the help of support vector machines. Conditional on each prepaid parameter set ***g*** in the basic grid, a number of the remaining parameters can be integrated out beforehand. First, conditional on grid point ***g***, we have for 50 predetermined coherences $C_{k}^{g}$ simulated accuracies and response time distributions (see Fig 11). The coherences of the observed data can be estimated solely using the observed accuracies using simple linear interpolation. The estimated coherence for stimulus (or condition) *i* is denoted as ${\hat{C}}_{i}$. Corresponding to each of the 50 coherences $C_{k}^{g}$ for grid point ***g***, there is a pair of corresponding simulated RT densities $p_{ic}^{g}\left(t\right)$ (with *c* = 1, 2). As before, $p_{ic}^{g}\left(t\right)$ is scaled to the [0, 3] seconds window, and we can use a combination of translating (estimating ${\hat{T}}_{er}$), scaling (estimating $\hat{D}$) and interpolating. Specifically, we first calculate $\hat{s}$ as the optimal time scalar to match data with the model on grid point ***g***:

$$\begin{array}{c} \hat{s}=\sqrt{\frac{\frac{1}{\left(4N-1\right)}\sum_{ij}{\left(t_{ij}-{\hat{\mu }}_{t}\right)}^{2}}{\frac{1}{4}\sum_{ic}\int p_{ic}^{g}\left(t\right){\left(t-\mu_{t}^{g}\right)}^{2}dt}}, \end{array}$$

in which

$$\begin{array}{ccc} {\hat{\mu }}_{t} & = & \frac{1}{4N}\sum_{ij}t_{ij}\\ \mu_{t}^{g} & = & \frac{1}{4}\sum_{ic}\int p_{ic}^{g}\left(t\right)tdt. \end{array}$$

This formula capitalizes on the fact that the variance of a distribution does not change when it is simply shifted to the right by a constant. Hence, the ratio of the model’s decision time variance (without *T*_*e*_*r*) and the observed total response time variance (presumably shifted with some *T*_*e*_*r*) is still an estimator of the squared scale factor between them. Using this information, we can estimate the optimal $\hat{D}$ and ${\hat{T}}_{er}$ for grid point ***g*** as follows:

$$\begin{array}{ccc} \hat{D} & = & \frac{D_{0}^{g}}{\hat{s}}\\ {\hat{T}}_{er} & = & {\hat{\mu }}_{t}-\hat{s}\mu_{t}^{g}, \end{array}$$

with $D_{0}^{g}$ being the optimal scaling diffusion constant used for optimal storage in the database. This gives us a final effective parameter vector of $\left(v^{\prime },\gamma^{\prime },\kappa^{\prime },{\hat{C}}_{1},{\hat{C}}_{2},{\hat{C}}_{3},{\hat{C}}_{4},\hat{D},{\hat{T}}_{er}\right)$. Note that the last 6 elements of this vector are estimates conditional on the grid point ***g*** = (*v*′, *γ*′, *κ*′).

Next, we have to determine the single optimal parameter set (and thus also the optimal *v*′, *γ*′, and *κ*′). For this we need an objective function that compares the model based PDFs with those of the data. For this purpose, we use a (symmetrized) chi-square distance based on a set of bin statistics. For each stimulus’ observed set of choice RTs, ***t***_*i*_ = (***t***_*i*1_, ***t***_*i*2_) (with ***t***_*i*1_ the RTs for option 1 and ***t***_*i*2_ for option 2), we calculate 20 data quantiles *q*_*u*_ (with *u* = 1, …, 20) at probability masses *m*_*i*_ = 0.05 ⋅ *i*. The set of quantiles is appended with one extra quantile *q*_0_ at *m*_0_ = 0.01 to have a more detailed representation of the leading edge of the distribution. Based on binning edges (0, *q*_0_, *q*_1_, …, *q*_20_, + ∞), we create 4 × 2 × 22 bin frequencies ${\hat{b}}_{icw}$ with *w* = 1, …, 22. The corresponding probability masses $m_{icw}^{g}$ can be easily extracted from the prepaid PDFs $p_{ic}^{g}\left(t\right)$ as well. Observed and theoretical quantities can then be combined in the a symmetrized chi-square distance:

$$\begin{array}{ccc} d(g,\left\{c_{ij},t_{ij}\right\}) & = & \sum_{icw}\frac{\left({\hat{b}}_{icw}-m_{icw}^{g}\right){}^{2}}{{\hat{b}}_{icw}+m_{icw}^{g}} \end{array}$$ (29)

This defines a distance between all grid points *g* in the database and any data set.

In the following paragraphs we will present three ways of using this distance to calculate LCA estimates, each a bit more complicated than the previous one (but also more accurate): ${\text{CHISQ}}_{\text{NN}}^{\text{Grid}}$, ${\text{CHISQ}}_{\text{BS}}^{\text{Grid}}$, ${\text{CHISQ}}_{\text{BS}}^{\text{SVM}}$.

First we will discuss the ${\text{CHISQ}}_{\text{NN}}^{\text{Grid}}$ variant. Here, the grid point closest to the data set (as measured by the symmetrized chi-square distance function) can be used as a first nearest neighbor estimate.

Second we discuss the ${\text{CHISQ}}_{\text{BS}}^{\text{Grid}}$ variant. Not all parameters are treated equally in the estimation procedure. The parameters *C*_*i*_, *D* and *T*_*er*_ are estimated conditionally on all grid points ***g*** and then the other parameters are estimated conditionally on ${\hat{C}}_{i}$, $\hat{D}$ and ${\hat{T}}_{er}$. Moreover, these parameters are chosen in such a way that a specific aspect of the data (e.g., proportion of choices for option 1) is fitted perfectly (i.e., the coherence is chosen to result in probabilities perfectly equal to the proportions observed in the data). This would be no problem for an infinite amount of data. However, for finite data, the major disadvantage of this way of working is that any errors induced in the precursor step are propagated through the estimation process for *v*′, *γ*′ and *κ*′. This is because for finite data, the observed accuracies will typically not exactly coincide with the accuracies provided by the best model estimates. As the estimates ${\hat{C}}_{i}$ are (on each grid point) exactly fit to the observed accuracy and consequently, the effective grid points will all have this exact accuracy. In this variant, we tackle this estimator bias by non-parametrically bootstrapping the data and repeating the nearest neighbor estimate for every bootstrapped dataset. Taking the mean of this set of estimates (a method known as bagging; [32]), gives us a more accurate estimate. Additionally, we now have a standard error of the estimate (and confidence interval).

Lastly, we discuss the ${\text{CHISQ}}_{\text{BS}}^{\text{SVM}}$ variant. If we apply the bootstrap procedure, it may turn out that the selected grid points as nearest neighbor are not very diverse (this may happen with large sample sizes). In such a situation, it can be worthwhile to use an interpolator. So we may learn a support vector machine based on the bin statistics of the few unique bootstraps grid points available, together with the best overall unique grid points. In this variant, we propose to use a training set of 100 grid points in total. The SVM can then be used as an approximation for the bin statistics in the space between the grid points and hence for the objective function. We subsequently minimize the approximative SVM based objective function for every bootstrap, using differential evolution (as has been outlined above for the other applications).

Obviously, the quality of the SVM based estimate is limited by the quality of the SVMs that are trained to learn the relation between parameters and statistics. In addition, the same SVMs are used for all bootstrap samples, which may introduce an unwanted distortion in the uncertainty assessment. To account for the systemic bias that might have been introduced by the SVMs, we will add some random noise to each bootstrap estimate. The amount of random deviation that is added equals the size of the prediction error of the SVM. In this way, low quality SVMs are prohibited of biasing all bootstraps in the same way. The uncertainty of the SVMs is now incorporated in the final bootstrapped results.

#### Test set

The test set is created by uniformly sampling parameters according to Eq 28. Input differences $\frac{v_{1}-v_{2}}{2}$ are chosen to produce typical accuracies of 0.6, 0.7, 0.8, and 0.9. As is done in [24], excessively long PDFs (with a maximum RT larger than 5000ms) and excessively short PDFs (with a range below 400ms) are removed from the test set. Apart from the fact that these PDFs are deemed unrealistic [24] for typical choice RT data, this part of the parameter space suffers from inherent poor parameter identifiability, with very large confidence intervals and less meaningful estimates as a consequence. Because the new parametrization analytically integrates out scale (i.e., *D*) (and also shift *T*_*er*_), and is positively unbounded in these dimensions, we can expand the test set to cover a broader range of distributions than the ones covered in [24]. To broaden the range of the test, the distributions are scaled with a random factor ranging from 0.2 to 5. We will use this broadened test set to determine the method’s accuracy and coverage.

#### Results accuracy

**Fig 13:**
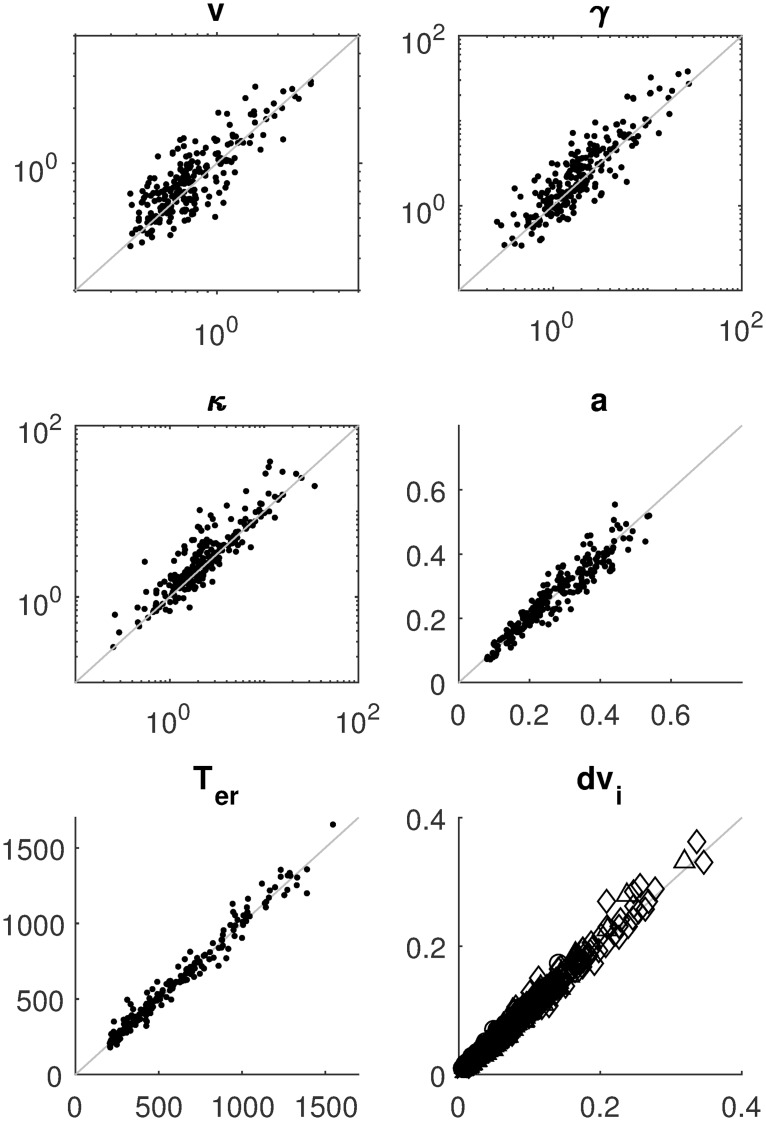
Recovery for the original parameters of the LCA model with *T*_obs_ = 1000 observation per stimulus. See Fig 4 for detailed information.

**Fig 14:**
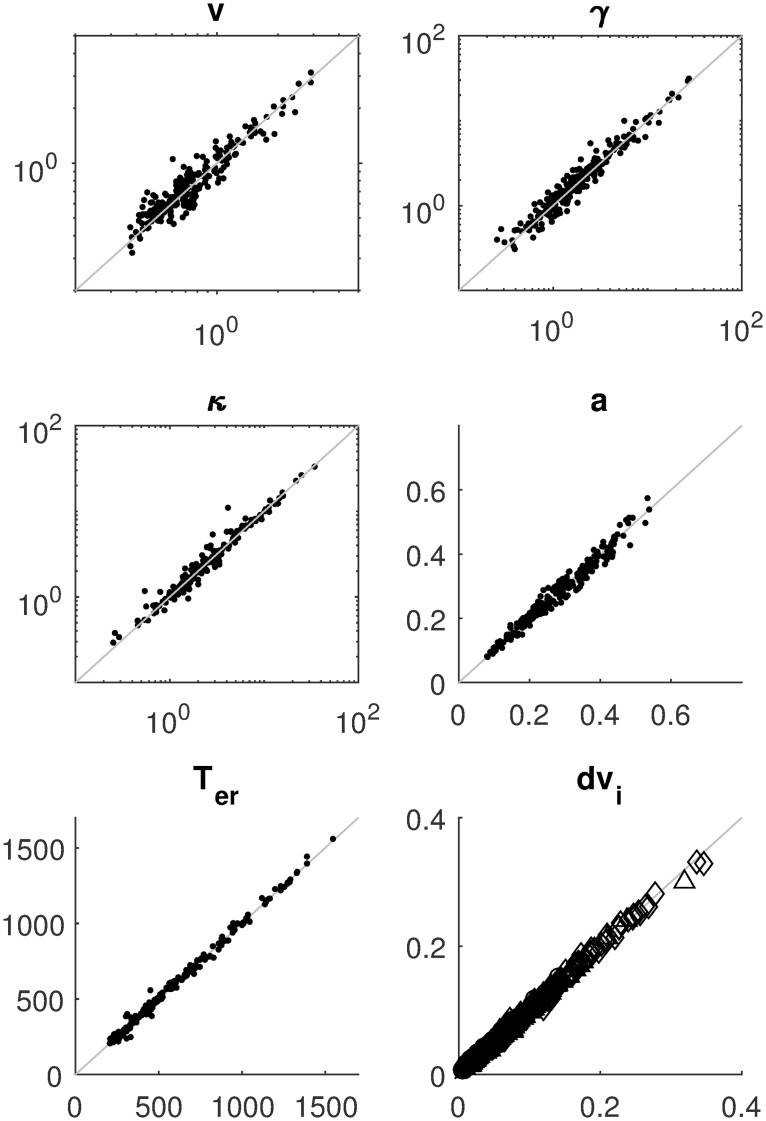
Recovery for the original parameters of the LCA model with *T*_obs_ = 10000 observation per stimulus. See Fig 4 for detailed information.

**Fig 15:**
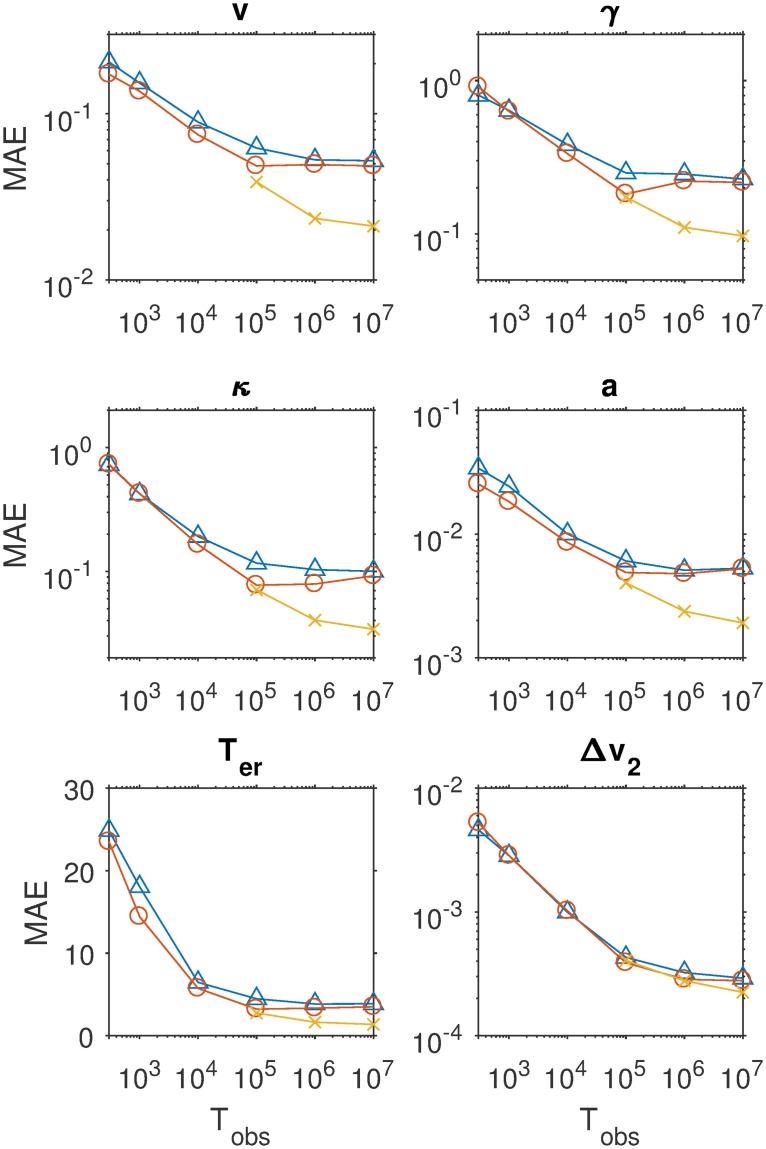
The MAE of the estimates of the parameters of the LCA as a function of sample size (abscissa) and for different methods. More details can be found in the caption of Fig 3.

**Fig 16:**
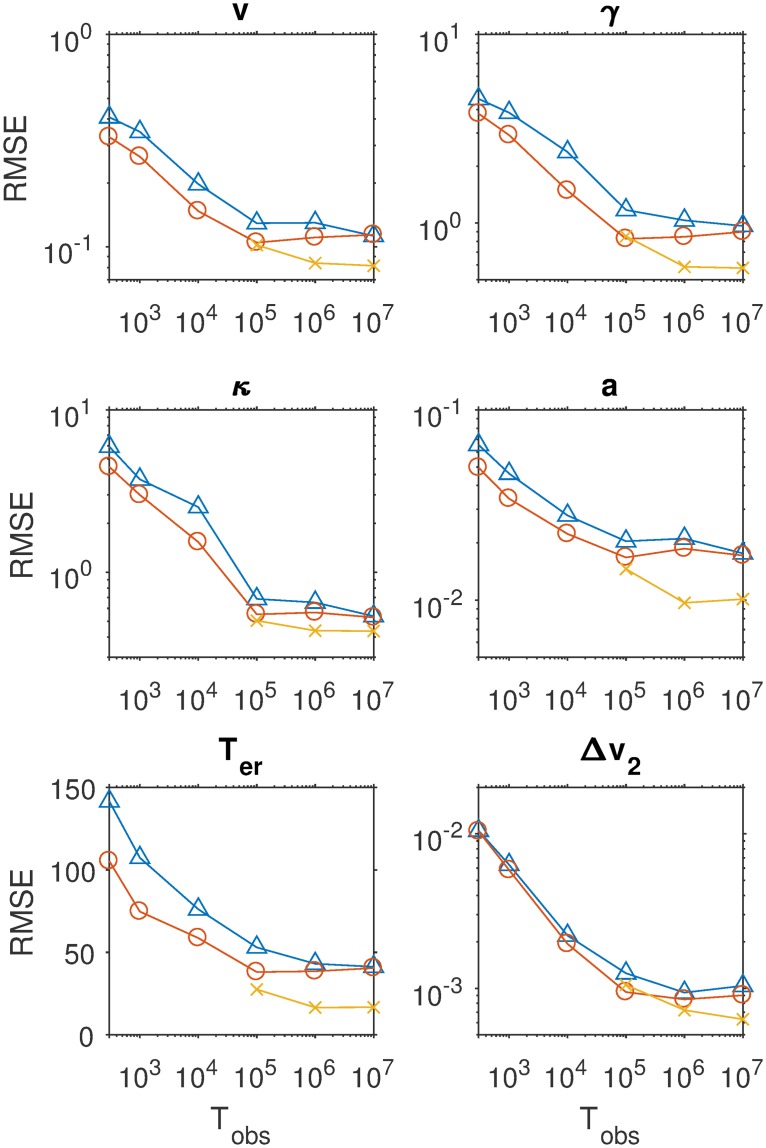
The RMSE of the estimates of the parameters of the LCA as a function of sample size (abscissa) and for different methods. More details can be found in the caption of Fig 3.

The recoveries of the original LCA parameters are displayed in Figs 4, 13 and 14. It can be concluded that for all sample sizes, recovery is acceptable, but it improves a lot for larger sample sizes. In all cases, the recovery is dramatically better than that reported in [24]. Figs 15 and 16 shows RMSE and MAE, respectively, as a function of sample size for three methods (for all parameters). It can be seen that accuracy improves for all parameters for the single best nearest neighbor and for the bootstrap method, until some point, after which it stabilizes or deteriorates. However, for the SVM based estimation, there is still considerable improvement for higher sample sizes.

#### Results coverage

**Fig 17:**
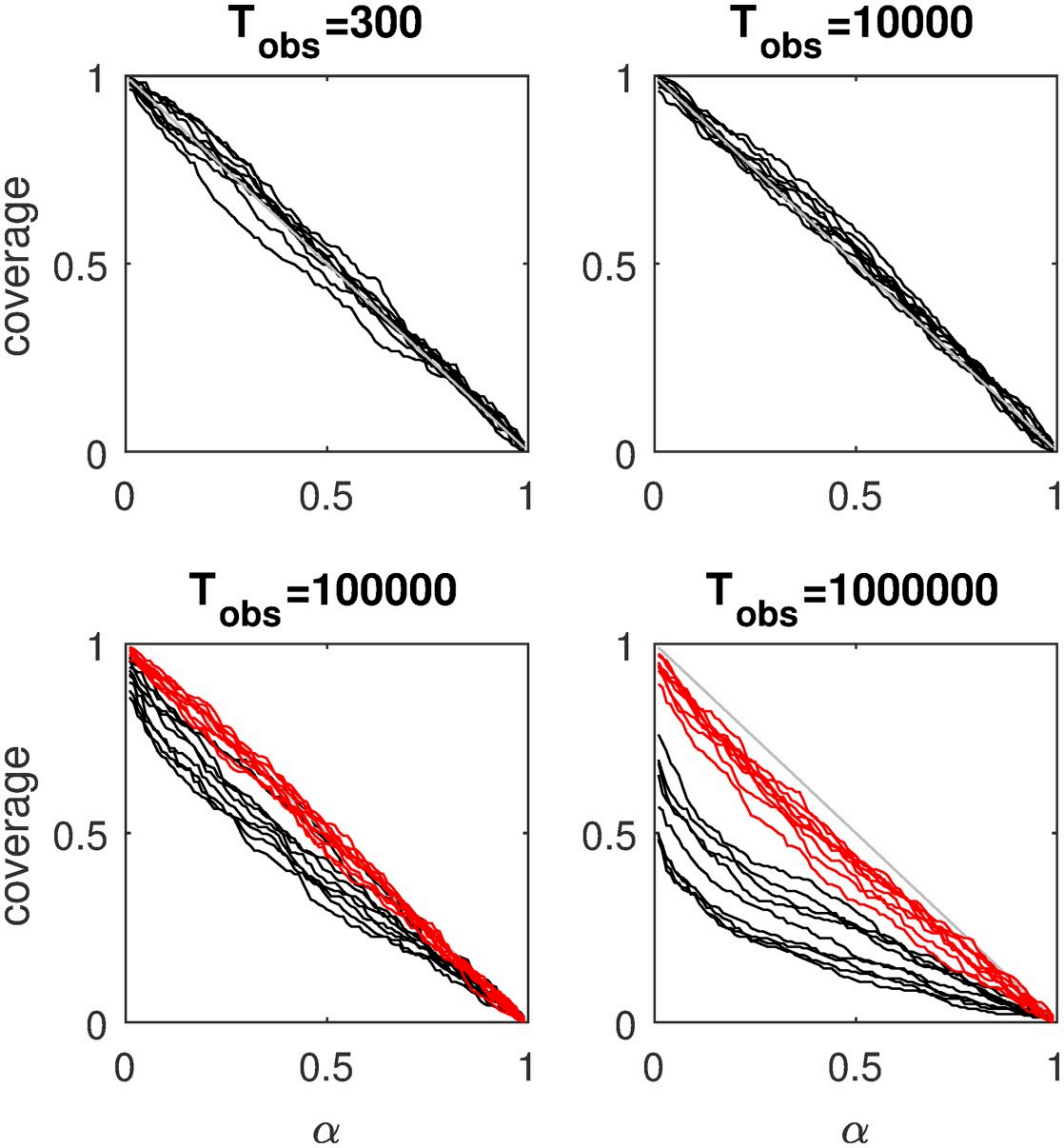
The coverage of LCA estimates for different number of observations *T*_obs_. Each line represents one of the nine LCA parameters and plots the fraction of estimates between the [*α*, 1 − *α*] quantiles of their bootstrapped confidence intervals. The closer the line to the second diagonal, the better the coverage. Black lines are the result of non-parametric bootstraps obtained through nearest neighbor estimates; red lines are the result of SVM enhanced estimates.

Fig 17 shows the coverages for different numbers of observations. Nearest neighbor bootstrap coverage seems to be adequate for sample sizes up to 10000; for higher sample sizes SVMs are needed to ensure good coverage.
